# Next‐Generation Image Sensors Based on Low‐Dimensional Semiconductor Materials

**DOI:** 10.1002/adma.202501123

**Published:** 2025-04-16

**Authors:** Yunxia Hu, Zhaoli Gao, Zhengtang Luo, Liang An

**Affiliations:** ^1^ Department of Chemical and Biological Engineering William Mong Institute of Nano Science and Technology and Hong Kong Branch of Chinese National Engineering Research Center for Tissue Restoration and Reconstruction The Hong Kong University of Science and Technology Hong Kong 999077 P. R. China; ^2^ Department of Mechanical Engineering The Hong Kong Polytechnic University Hong Kong 100872 P. R. China; ^3^ Department of Biomedical Engineering The Chinese University of Hong Kong Hong Kong 999077 P. R. China

**Keywords:** image sensors, low‐dimensional semiconductor materials, optical memory devices, optical synaptic devices, photodetectors

## Abstract

With the rapid advancement of technology of big data and artificial intelligence (AI), the exponential increase in visual information leads to heightened demands for the quality and analysis of imaging results, rendering traditional silicon‐based image sensors inadequate. This review serves as a comprehensive overview of next‐generation image sensors based on low‐dimensional semiconductor materials encompassing 0D, 1D, 2D materials, and their hybrids. It offers an in‐depth introduction to the distinctive properties exhibited by these materials and delves into the device structures tailored specifically for image sensor applications. The classification of novel image sensors based on low‐dimensional materials, in particular for transition metal dichalcogenides (TMDs), covering the preparation methods and corresponding imaging characteristics, is explored. Furthermore, this review highlights the diverse applications of low‐dimensional materials in next‐generation image sensors, encompassing advanced imaging sensors, biomimetic vision sensors, and non‐von Neumann imaging systems. Lastly, the challenges and opportunities encountered in the development of next‐generation image sensors utilizing low‐dimensional semiconductor materials, paving the way for further advancements in this rapidly evolving field, are proposed.

## Graphical abstract

Low‐dimensional semiconductor materials are promising candidates for photosensitive components in next‐generation image sensors. This review offers a thorough and timely examination of novel image sensors, covering their working principles, intriguing materials categorized into four main groups, and advanced imaging applications. Additionally, it delves into the roadmap for next‐generation image sensors, exploring future opportunities and challenges in the field.

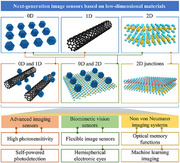

## 1 Introduction

Image sensors have been instrumental in capturing and converting visual information into electrical signals, allowing for the interpretation, analysis, and comprehension of our surroundings.^[^ ^1^, ^2^, ^3^ ^]^ Nowadays, traditional commercial image sensors based on charge‐coupled device (CCD) and complementary metal‐oxide semiconductor (CMOS) architectures have been ubiquitously applied in diverse applications including consumer electronic products and industrial applications.^[^ ^4^, ^5^ ^]^ However, the 3D silicon materials applied in conventional image sensors show high stiffness, low fracture roughness, high modulus, and narrow bandgap (400–1100 nm), restricting the development of next‐generation imaging functions such as ultrahigh photosensitivity, zero electrical consumption, flexibility, biomimetic hemispherical imaging, and neuromorphic vision.^[^ ^6^, ^7^ ^]^ Hence, there is significant interest in exploring novel materials and device structures to drive the advancement of next‐generation image sensors.

In the past few years, a variety of functional low‐dimensional materials^[^ ^8^, ^9^ ^]^ including 0D quantum dots (QDs),^[^ ^10^ ^]^ 1D nanowires/nanorods,^[^ ^11^ ^]^ 2D sheets,^[^ ^12^, ^13^ ^]^ and their hybrid (0D/2D, 1D/2D, and 2D/2D heterostructures)^[^ ^14^ ^]^ have been investigated for image sensors. Due to the unique structure with at least one dimension at the nanoscale level and large surface‐volume ratios,^[^ ^15^, ^16^ ^]^ low‐dimensional materials showcase exceptional light‐matter interaction and excellent mechanical flexibility, making them significantly superior to their bulk counterparts.^[^ ^17^, ^18^ ^]^ In terms of dimensions, 0D QDs, such as indium arsenide (InAs),^[^ ^19^ ^]^ lead sulfide (PbS),^[^ ^20^ ^]^ and indium phosphide (InP)^[^ ^21^ ^]^ colloidal quantum dots (CQDs), offer size‐tunable bandgaps, cover broad spectral ranges and demonstrate exceptional light absorption capabilities. Likewise, 1D nanowires, including Zn_2_GeO_4_ microwires^[^ ^22^ ^]^ and CH_3_NH_3_PbI_3_ nanowires,^[^ ^23^, ^24^ ^]^ have been developed for devices integration and flexible photodetectors. 2D materials such as graphene,^[^ ^25^ ^]^ transition metal dichalcogenides (TMDs),^[^ ^26^ ^]^ hexagonal boron nitride (h‐BN),^[^ ^27^ ^]^ and black phosphorus (BP)^[^ ^28^ ^]^ exhibit ultrathin thickness and remarkable light‐matter interaction. Moreover, the fabrication of heterostructures by combining various types of low‐dimensional materials^[^ ^29^, ^30^ ^]^ enables the manipulation of light absorption, expanding the spectral range and improving light sensitivity. Consequently, there is urgent need for systematically summarizing low‐dimensional materials for next‐generation image sensors.

Based on the combined effect of material properties and device structures, imaging applications using low‐dimensional semiconductor materials is categorized into three groups:^[^ ^31^, ^32^, ^33^, ^34^ ^]^ 1) advanced image sensor with high photoelectric conversion efficiency,^[^ ^35^, ^36^ ^]^ broadband spectral range,^[^ ^37^, ^38^ ^]^ or zero consumption.^[^ ^39^, ^40^ ^]^ These image sensors are accomplished through various means, such as combining PbS colloidal QDs with CMOS devices,^[^ ^41^ ^]^ selecting materials with suitable bandgaps,^[^ ^42^ ^]^ and designing asymmetric contacts in a two‐terminal photodetectors.^[^ ^39^ ^]^ 2) Biomimetic vision sensors with flexible or hemispherical imaging functions utilizing the ultrathin thickness character and mature synthesized method on several substrates of low‐dimensional materials,^[^ ^3^, ^43^ ^]^ such as near‐infrared (NIR) In_2_Se_3_/MoS_2_ heterojunctions for a flexible 10 × 10 devices array^[^ ^44^ ^]^ and a hemispherical perovskite nanowire array retina.^[^ ^45^ ^]^ 3) Non‐von‐Neumann imaging systems for image sensing and learning functions. The presence of artificial defects in low‐dimensional materials leads to the trapping and releasing of charges, which is driven by the combination of light and electricity to achieve synaptic plasticity.^[^ ^46^, ^47^ ^]^ Optoelectronic devices utilizing floating‐gated structures are capable of achieving memory functions through precise control of the on‐state and off‐state operations, which have been demonstrated for non‐von‐Neumann imaging systems.^[^ ^48^, ^49^ ^]^

**Figure 1:**
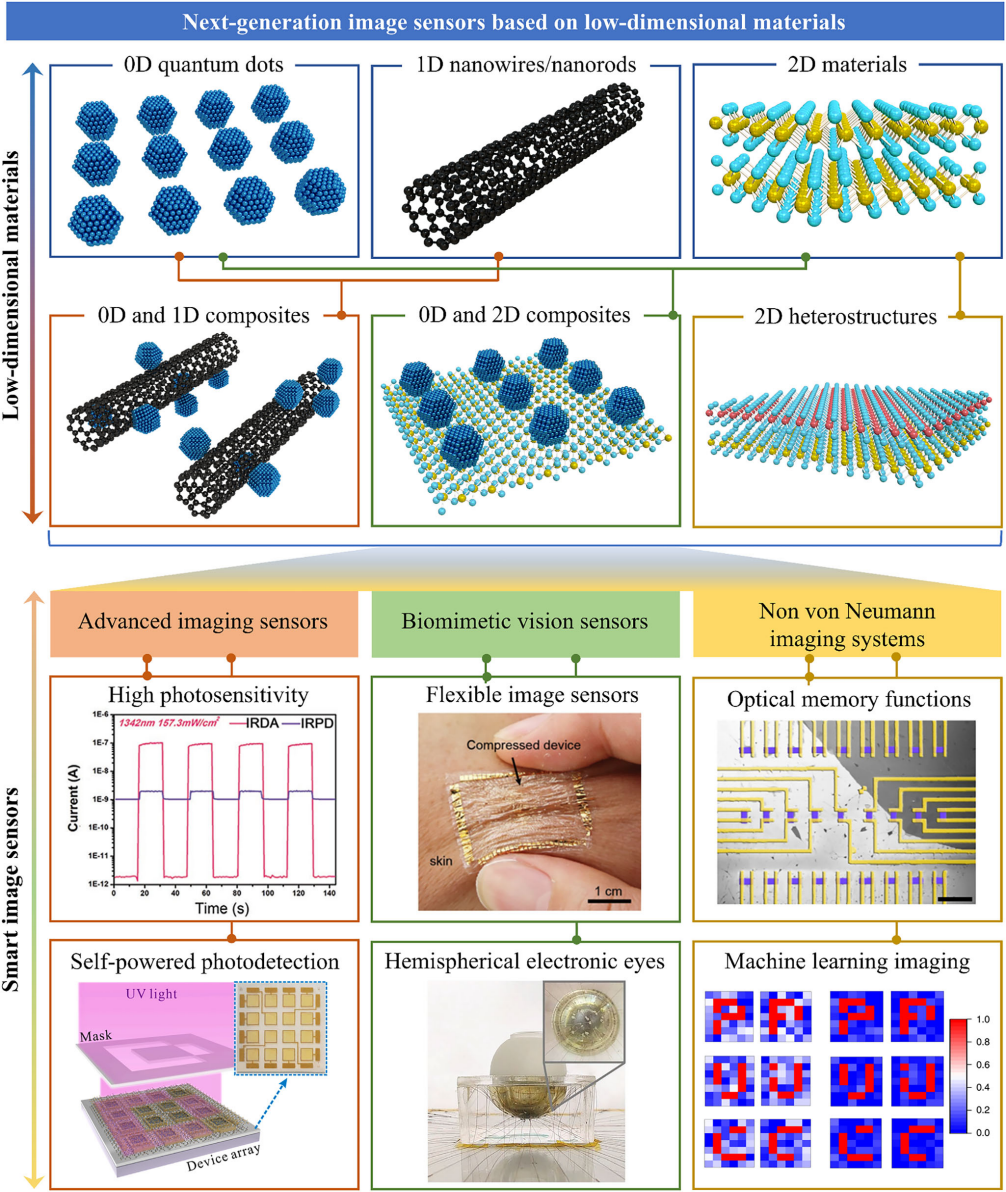
Overview of low‐dimensional materials and applications. The next‐generation image sensors based on low‐dimensional materials including 0D QDs, 1D nanowires/nanorods, 2D materials, and hybrid materials, which have exhibited excellent electrical and optical properties, and enormous prospects in imaging applications. Reproduced with permission.^[^ ^50^ ^]^ Copyright 2020, WILEY‐VCH Verlag GmbH. Reproduced with permission.^[^ ^51^ ^]^ Copyright 2022, Elsevier Ltd. Reproduced with permission.^[^ ^52^ ^]^ Copyright 2021, WILEY‐VCH Verlag GmbH. Reproduced with permission.^[^ ^45^ ^]^ Copyright 2020, Springer Nature Limited. Reproduced with permission.^[^ ^53^ ^]^ Copyright 2018, Springer Nature Limited. Reproduced with permission.^[^ ^54^ ^]^ Copyright 2019, Springer Nature Limited.

The review begins by introducing the working principles of image sensors and mechanisms of photodetection, providing a foundation for understanding their operation. Subsequently, image sensors based on low‐dimensional semiconductor materials are systematically classified and detailly introduced according to the material dimensions (0D quantum dots, 1D nanowires/nanorods, 2D materials, 0D/1D composites, 0D/2D composites, as well as 2D heterostructures), as summarized in **Figure** **1**. By designing and constructing single‐pixel or multi‐pixel image sensors based on these low‐dimensional semiconductor materials, their applications have been explored and also summarized in Figure 1 including advanced imaging sensors with high photosensitivity^[^ ^50^ ^]^ and self‐powered photodetection,^[^ ^51^ ^]^ biomimetic vision sensors including flexible image sensors^[^ ^52^ ^]^ and hemispherical electronic eyes,^[^ ^46^ ^]^ as well as non von Neumann imaging systems with optical memory functions^[^ ^53^ ^]^ and machine learning imaging.^[^ ^54^ ^]^ Currently, most low‐dimensional semiconductor materials, particularly TMDs, are produced through mechanical exfoliation^[^ ^53^ ^]^ and chemical vapor deposition (CVD) methods,^[^ ^26^ ^]^ which limits the resolution and integration of image sensors. The ultrathin nature of these materials leads to low light absorption,^[^ ^39^ ^]^ resulting in weak photosensitivity and high energy efficiency. Furthermore, effective integration with flexible substrates and artificial intelligence (AI) remains a challenge, along with the demands of miniaturization, which hinders the advancement of next‐generation image sensors. These challenges also present opportunities. There is increasing focus on developing high‐quality, wafer‐scale low‐dimensional semiconductor materials, designing multifunctional devices that achieve high sensitivity and low energy consumption, and implementing strategies for integration. Particularly promising are the opportunities arising from the integration of AI and the development of flexible, wearable technologies, which could significantly enhance intelligent imaging systems and their practical applications.

## 2 Working Principles and Mechanisms for Image Sensors

The implementation of image sensors based on low‐dimensional materials, which convert optical images into digital signals, relies on similar underlying working principles. Each device in these two types has an individual operation mechanism depending on the device architecture such as two‐terminal photodetectors, phototransistors, defects‐induced optical synaptic devices, and floating‐gate optoelectronic memory devices.

### 2.1 Single‐Pixel Imaging and Multi‐Pixel Imaging

**Figure 2:**
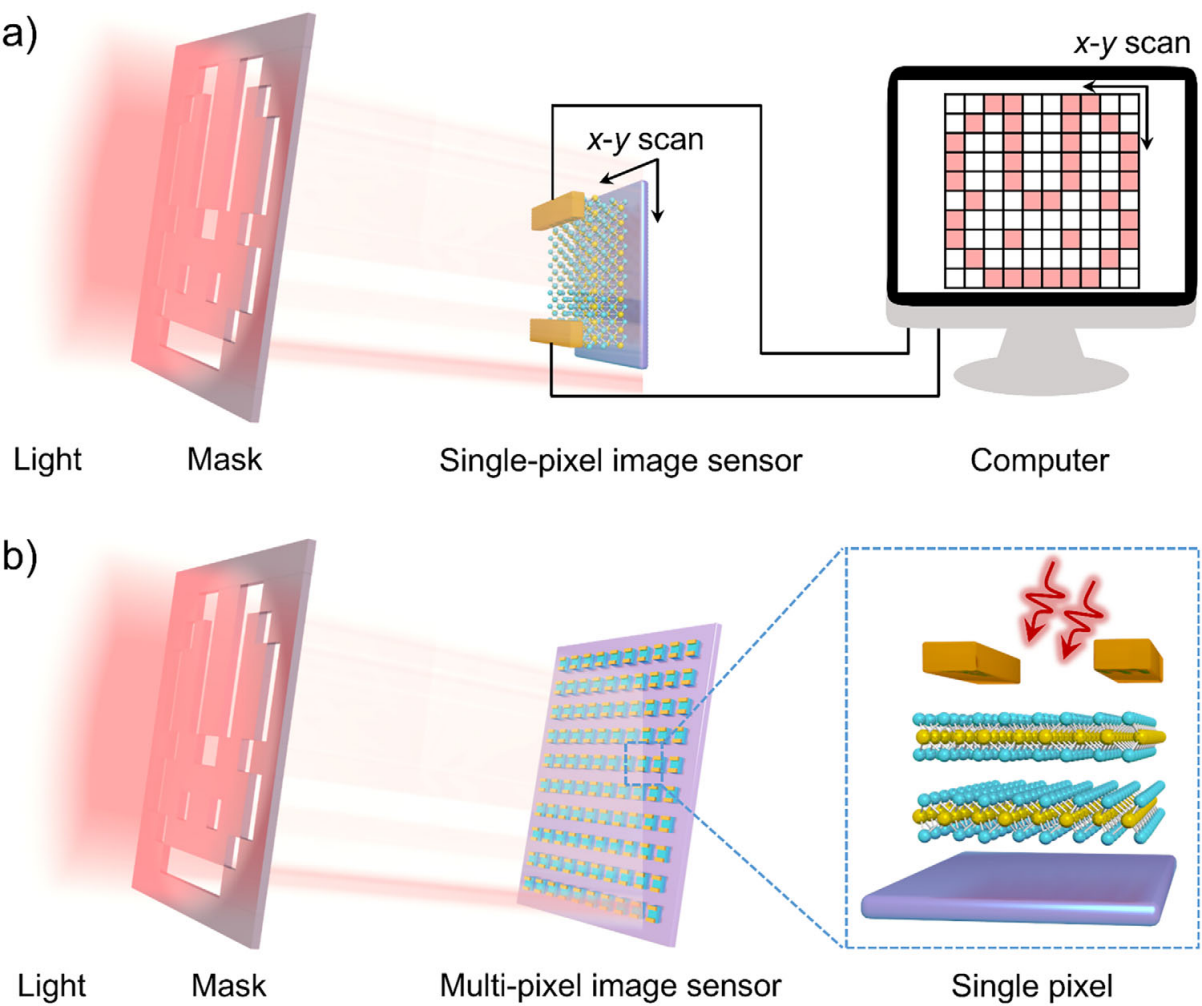
Work principle of the imaging process. The working principle of imaging through a) the single‐pixel mode. The light illuminates through a mask onto a single photodetector to capture images by the relative movement of the patterned light and the photodetector in two directions (x, y). b) based on the multi‐pixel image sensor. The light illuminates the devices array through a patterned mask on devices array, resulting in the formation of the image.

The working principles of image sensors refer to the mechanism of how patterned light illuminates onto the image sensor to ultimately realize a patterned electrical image. The single‐pixel imaging and multi‐pixel imaging are two main working principles, which are shown in **Figure** **2**. The single‐pixel imaging technique based on a single photodetector relies on the relative movement between the light source and the image sensor, which influences the image quality including spatial resolution, sampling accuracy, and the signal‐to‐noise ratio (SNR). Precise and stable movement is essential due to that the misalignment can lead to artifacts and blurring and rapid movement may introduce motion blur if not synchronized properly. Additionally, single‐pixel systems are vulnerable to unintended movements due to their reliance on sequential scanning, restricting the application in dynamic scenes and real‐time imaging of fast‐moving subjects.^[^ ^55^ ^]^ Despite these challenges, the single‐pixel imaging system offers a simplified and cost‐effective setup compared to complex and expensive CCD or CMOS image sensors, which is applied in situations where multi‐pixel imaging systems are limited, such as specialized terahertz imaging,^[^ ^56^, ^57^ ^]^ infrared imaging,^[^ ^58^ ^]^ low‐light conditions,^[^ ^59^ ^]^ and imaging with time‐based depth resolution.^[^ ^60^ ^]^

Figure 2a shows the schematic diagram of the single‐pixel imaging system, utilizing patterned light illumination and a single photodetector to capture images by scanning in two directions (x, y). The patterned mask determines the image shape, and the light wavelength or intensity‐dependent photocurrent leads to image color or contrast, enables imaging identification. Moreover, single‐pixel imaging exhibits outstanding optoelectronic performance, including a broad spectrum range, photoresponse dependent on light intensity, rapid response speed, and high‐resolution imaging results.^[^ ^61^, ^62^ ^]^ In a recent report, a typical single‐pixel image system based on a Bi_2_Se_3_/Bi_2_Se_x_O_y_ heterostructure photodetector is constructed.^[^ ^63^ ^]^ Complex patterns with high resolutions and large sizes are carried out by this single‐pixel imaging system. The vertical‐lateral Bi_2_Se_3_/Bi_2_Se_x_O_y_ heterostructure exhibits excellent imaging properties, characterized by a high SNR and superior stability. Imaging under low‐light conditions holds immense significance in imaging applications including biological imaging, night vision, and astronomy. High‐resolution images under weak‐light UV illumination are achieved by a single‐pixel imaging system based on a 2D perovskite single crystal with responsivity of 2.22·A W^−1^ under 5.49 nW·cm^−2^ of UV illumination.^[^ ^59^ ^]^ Additionally, a single‐pixel image sensor based on 2D Bi_2_O_2_Se/In_2_S_3_ heterostructure with a unilateral depletion band design has been presented to exhibit ultraweak light imaging capabilities even under 0.5 pW of 405 nm light.^[^ ^64^ ^]^ These results demonstrate that single‐pixel imaging is an effective solution for low‐light imaging and low‐dimensional materials show great potential in practical imaging applications. However, a notable drawback is the relatively long imaging time, which limits its application in capturing moving objects in real‐world environments.

The multi‐pixel imaging uses an array of photodetectors to capture multiple pixels simultaneously, leading to fast image acquisition and high image quality.^[^ ^2^, ^65^ ^]^ Figure 2b depicts the setup of a multi‐pixel imaging system that utilizes low‐dimensional materials.^[^ ^66^ ^]^ The light illuminates the devices array through a patterned mask, forming an electrical image. Each pixel contains a photosensitive device that detects under different illumination conditions, converting light into electrical signals. This multi‐pixel imaging process offers rapid image acquisition and superior image quality. For example, a multi‐pixel image sensor with a vertical structure has been fabricated, utilizing a perovskite photodetector array.^[^ ^66^ ^]^ The crossbar configuration of the image sensor has simplified the electrode fabrication and maintained the excellent photodetection properties of a large linear dynamic range (*LDR*) of 50.35 dB, with a maximum detectivity of 4.2 × 10^11^ Jones and responsivity of 7 A·W^−1^. The lateral photosensitive semiconductor channel is another device configuration in image sensors.^[^ ^67^, ^68^ ^]^ An image sensor with 8 × 8 pixels based on a bilayer MoS_2_ film, realizing the ability to distinguish light wavelengths of 638, 532, and 405 nm at a consistent light intensity.^[^ ^69^ ^]^ Multi‐pixel image sensors composed of the photodetector arrays offer time‐saving imaging processes and excellent photodetection properties. Currently, there is a rising interest in research toward attaining high resolution, outstanding photodetection, multifunctional imaging capabilities, and flexibility using multi‐pixel imaging technology.

With the rapid development of AI, the integration of computational algorithms with imaging functions has attracted more and more attention to address unique challenges and unlocking new capabilities.^[^ ^70^, ^71^, ^72^ ^]^ For single‐pixel imaging systems, compressed sensing reduces the required measurements by 50–90% through sparsity exploitation, enabling faster acquisition without sacrificing resolution in terahertz^[^ ^56^ ^]^ and infrared imaging.^[^ ^61^ ^]^ Deep learning such as convolutional neural networks reconstructs high‐fidelity images from sparse or noisy data, improving SNR by 5–10 dB in low‐light conditions and optimizing illumination patterns for dynamic scenes by adaptive sampling dynamically.^[^ ^55^ ^]^ For multi‐pixel systems, non‐local algorithms with denoising and super‐resolution networks enhance image clarity and resolution, mitigating noise and pixel limitations in conventional sensors.^[^ ^20^ ^]^ These innovations bridge the gap between theoretical performance and practical utility, enabling single‐pixel systems to rival multi‐pixel systems in specialized applications while empowering multi‐pixel systems to achieve unprecedented speed and accuracy.

In summary, single‐pixel imaging provides high‐resolution and low‐noise performance by using a single high‐sensitivity detector and sequential scanning, achieving excellent SNR for specific wavelengths like terahertz and infrared. However, the slow imaging speed of single‐pixel imaging makes it less suitable for dynamic or real‐time applications. In contrast, multi‐pixel imaging enables rapid, parallel data capture ideal for real‐time scenarios, but it faces challenges such as higher noise levels and increased complexity in large‐scale sensor fabrication.

### 2.2 Functional Devices and Mechanisms

**Figure 3:**
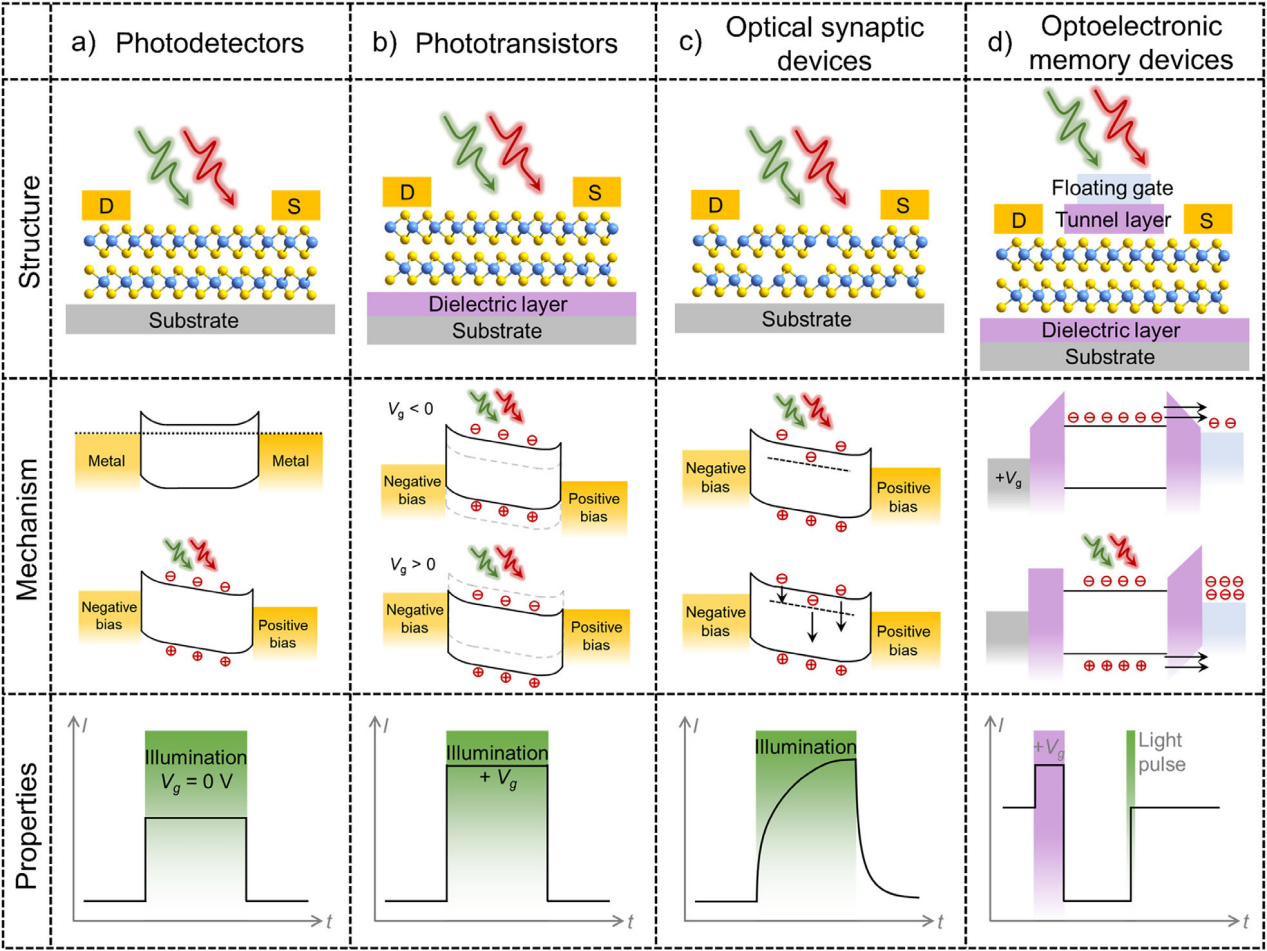
Optoelectronic devices in image sensors. Schematic diagram of the device structure, energy band variation under illumination, and the corresponding photoresponse curves for a) two‐terminal photodetectors, which convert light to electrical signal; b) phototransistors, in which light is used to amplify the signal of the transistor; c) optical synaptic devices, which use light to transmit and process information, enabling neuromorphic computing by utilizing defects; d) optical memory devices that use light to read or write data. Several key performance metrics are defined as follows. For two‐terminal photodetectors and phototransistors: 1) photocurrent (*I*_ph_), the difference in current between illuminated and dark conditions; 2) photoresponsivity (*R*), the ratio of photocurrent to the illuminated light power; 3) external quantum efficiency (*EQE*), the fraction of photogenerated electron‐hole pairs that are captured by the electrodes relative to the number of incoming photons; and 4) specific detectivity (*D* ^*^), which measures the SNR produced per unit of irradiation power under a specified bandwidth and area. For optical synaptic devices: 1) the pair‐pulse‐facilitation (PPF) index, the ratio of the current amplitude of the second light pulse (*A*_2_) to that of the first light pulse (*A*_1_); 2) short‐term plasticity (STP), the storage of current states ranging from a few seconds to a few minutes; and 3) long‐term plasticity (LTP), which maintains current states from minutes to years. In the case of optoelectronic memory devices: 1) the on/off ratio, representing the ratio of on‐state current to off‐state current; and 2) retention time, which indicates how long the on‐state current can be maintained.

The device architecture derived from low‐dimensional materials engenders functional properties, which is a crucial factor in defining the application domains of image sensors.^[^ ^73^ ^]^ By amalgamating the advantages of low‐dimensional materials with functional device structures, the image sensors have showcased diverse and versatile applications in intelligent image detection. This section provides in‐depth analysis for the photoresponse mechanism including generation, transport, and recombination processes of photo‐generated carriers in low‐dimensional semiconductor materials by combining experiments results and theoretical calculations. Then the device structures based on low‐dimensional semiconductor materials in image sensors are classified into four categories: two‐terminal photodetectors, phototransistors, defects‐induced optical synaptic devices, and floating‐gate optoelectronic memory devices, shown in **Figure** **3**. Figure 3 summarizes the reported device type and comparison with structural design, photodetection mechanisms, and time‐dependent photoresponse properties.

#### 2.2.1 Photoresponse Mechanism

The photoresponse process based on low‐dimensional semiconductor materials involves several key steps: under illumination, the light with energy (*hυ*) higher than the bandgap of materials (*E*_g_) can be absorbed to excite electrons from the valence to the conductance band, leading to the generation of additional electron‐hole pairs.^[^ ^74^, ^75^ ^]^ Then the photogenerated electron‐hole pairs are divided by the external source‐drain bias (*V*_ds_) or built‐in electrical fields, inducing the photogenerated electrons and holes drift in opposite directions to increase current under light (*I*_light_) compared to darkness (*I*_dark_).^[^ ^76^ ^]^ The energy band structures of materials result in a photoresponse that varies with wavelength, and higher light intensity generates more photogenerated electron‐hole pairs. The Se/InSe heterojunction exhibits a maximum responsivity of 35 mA·W⁻¹ at 460 nm, with an increased photocurrent response as light intensity is enhanced.^[^ ^77^ ^]^ In addition to the application of external voltage, the built‐in electric field of the photodetector can separate the photogenerated electron‐hole pairs. By designing an InSe‐based asymmetric Schottky junction photodetector, self‐powered photodetection across the ultraviolet (UV) to visible spectrum is achieved.^[^ ^39^ ^]^

In the process of photoresponse generation, the transfer and recombination of photogenerated carriers also exist simultaneously. Due to several dimensions of low‐dimensional semiconductor materials are reduced from bulk to atomic level, Coulomb interactions are enhanced compared to bulk materials, resulting in the generation of excitons with electrons and holes tightly bound together. Therefore, the transfer and combination processes of photogenerated carriers in low‐dimensional semiconductor materials are influenced.^[^ ^78^ ^]^ Non‐equilibrium photogenerated carriers typically experience several relaxation processes, such as rapid thermalization through carrier‐carrier scattering, cooling to the band edges via phonon interactions, and electron‐hole recombination, either directly or with the help of defects or phonons.^[^ ^79^ ^]^ Ultrafast laser spectroscopy has unveiled the photocarrier dynamics in two‐dimensional materials, demonstrating that photocarriers typically relax to the band edges and often form excitons within about 1 ps before recombination. Additionally, excitons can be directly generated through optical excitation at the excitonic resonance.^[^ ^80^ ^]^ Moreover, ultrafast charge transfer occurring within 50 fs from the MoS_2_ layer to the WS_2_ layer in MoS_2_/WS_2_ heterostructures has been demonstrated using photoluminescence (PL) mapping and femtosecond pump‐probe spectroscopy.^[^ ^81^ ^]^ Furthermore, recombination centers like sulfur vacancies in WS_2_ create defect trap states that hinder carrier recombination and extend the photoresponse time.^[^ ^82^ ^]^ By incorporating these insights into the photoresponse mechanism, photodetection properties can be optimized for practical applications in next‐generation image sensors.

#### 2.2.2 Two‐Terminal Photodetectors

As shown in Figure 3a, the two‐terminal photodetectors include semiconductor channels and two electrodes, creating a metal‐semiconductor‐metal (MSM) configuration.^[^ ^83^, ^84^ ^]^ The photogenerated electron‐hole pairs in two‐terminal photodetectors are separated by external bias based on the photoconductive mechanism or built‐in electrical fields via the photovoltaic mechanism for photogenerated carriers to produce photocurrent.^[^ ^85^, ^86^ ^]^ Figure 3a illustrates the device structure, energy band, and time‐dependent photoresponse properties. The increase in current under illumination, known as the photocurrent (*I*_ph_) is calculated to evaluate the photoresponse capability:

(1) $$I_{\mathrm{ph}}=I_{\mathrm{light}}-I_{\mathrm{dark}}$$

Various figures of merit have been defined to assess the performance of photodetectors across different sizes and lighting scenarios.^[^ ^12^, ^87^ ^]^ Photoresponsivity (*R*) and external quantum efficiency (*EQE*) are crucial metrics for evaluating the effectiveness of photoelectric conversion. Photoresponsivity, which quantifies the photocurrent relative to the light power reaching the channel region in the photodetector, is defined as:

(2) $$R=\frac{I_{\mathrm{ph}}}{P}\;\left(\frac{A}{W}\right)$$

where *P* is the light power equal to product of light power density (*P*_i_) and illuminated channel area (*A*). And the *EQE* represents the proportion of photogenerated carriers effectively captured by electrodes (*N*_C_) to enhance the current, compared to the number of incident photons (*N*_I_), and can be computed using the subsequent formula:^[^ ^12^ ^]^

(3) $$\mathrm{EQE}=\frac{N_{C}}{N_{I}}=\frac{\mathrm{hc}}{e\lambda }\;R$$

where *e* is the electron charge (1.602176634 × 10^−19^ C), *h* is the Planck constant (6.62606957 × 10^−34^ m^2^kg s^−1^), *c* is the light speed (3 × 10^−8^ ms^−1^), and *λ* (nm) is the wavelength of the incidence light. Both the photoresponsivity and external quantum efficiency vary with wavelength and light intensity because of the distinct energy bands and light absorption characteristics inherent to materials.

Sensitivity, a critical photodetector parameter, is evaluated using specific detectivity (*D* ^*^) excluding the influence of the bandwidth, geometry, and the area of device, which is defined as:

(4) $$D^{*}=\frac{{\left(A\cdot \mathrm{BW}\right)}^{1/2}}{\mathrm{NEP}}\;\;\left(\mathrm{cm}\cdot {\mathrm{Hz}}^{1/2}\cdot W^{-1}\left(\mathrm{Jones}\right)\right)$$

where *A* is the active area, *BW* is the frequency bandwidth. Noise equivalent power (*NEP*) represents the minimum light signal power that a photodetector can detect or differentiate from the total noise, which includes ambient noise, internally generated noise, and other sources. The *NEP* (W·Hz^−1/2^) is equal to *i*_N_/*R*, where *i*_N_ is noise current at 1 Hz bandwidth with units of A·Hz^−1/2^. The larger specific detectivity value means better sensitivity of the photodetector. Response time, including rise time (*τ*_r_) and decay time (*τ*_d_), measures photoresponse sensitivity to light changes. Comparing photodetectors across various low‐dimensional materials and device structures involves evaluating these metrics under diverse light conditions.

The linear relationship between photoresponse and incident light power lies in the predictability and reliability of a photodetector, which is defined as the *LDR*. The amount of the *LDR* is expressed by the following equation:^[^ ^88^ ^]^

(5) $$\mathrm{LDR}=20\log\;\frac{I_{\max}}{I_{\min}}=20\log\frac{P_{\max}}{P_{\min}}$$

where *I*_max_ (*P*_max_) and *I*_min_ (*P*_min_) represent the maximum and minimum values of the photocurrent (light intensity) within the linear range of the light intensity‐dependent photocurrent curve. This linearity indicates that as the intensity of the incoming light increases, the photocurrent increases proportionally, allowing for accurate measurements of light intensity.

#### 2.2.3 Phototransistors

Phototransistors, shown in Figure 3b, are light‐sensitive three‐terminal devices structured like field‐effect transistors (FETs). They exhibit high gains and low dark currents that are controlled by gate voltages, making them promise for optoelectronic applications.^[^ ^89^, ^90^ ^]^ Figure 3b schematically shows the typical FET device structure. The low‐dimensional materials as semiconductor channels are isolated with the gate voltage by dielectric materials.^[^ ^91^ ^]^ The conductance is adjusted by altering energy bands and carriers through varying illumination and gate voltages.^[^ ^92^ ^]^ In n‐type semiconductors, positive gate voltages decrease energy bands, prompting electron accumulation at the interface, while negative gate voltages repel electrons, raising energy bands.^[^ ^93^ ^]^ The synergistic effects of positive gate voltages and light exposure on semiconductor carriers result in a significant contrast in current levels between illuminated and dark conditions, enhancing the photodetection capabilities. Various approaches have been proposed to boost the photodetection capabilities of phototransistors constructed from low‐dimensional materials. For instance, a p‐n junction consisting of a WSe_2_ phototransistor to enable the electrostatic tuning of photocarrier population and channel width.^[^ ^94^ ^]^ Additionally, selecting suitable dielectric layers like hexagonal boron nitride (hBN),^[^ ^95^ ^]^ aluminum oxide,^[^ ^96^ ^]^ or hafnium oxide,^[^ ^97^ ^]^ enhance the electric field effect to meet specific design criteria.

#### 2.2.4 Defects Induced Optical Synaptic Devices

The biological synapse acts as the fundamental unit in the nervous system, enabling signal transmission and processing through neurotransmitter release across the synaptic cleft.^[^ ^98^, ^99^ ^]^ The excitatory postsynaptic current or inhibitory postsynaptic current means increase or decrease in the synaptic weight, respectively.^[^ ^100^, ^101^ ^]^ Inspired by the biological synapses, optical synaptic devices have been proposed using light pulses as neurotransmitters and devices as postsynaptic membranes.^[^ ^47^, ^102^ ^]^ Figure 3c illustrates the defect‐induced synaptic devices. By strategically introducing defects like doping,^[^ ^103^ ^]^ vacancies,^[^ ^46^, ^104^ ^]^ or native oxide layers,^[^ ^47^, ^105^ ^]^ this method alters carrier dynamics within the energy band of low‐dimensional materials to replicate the functionality of biological synapses. As carriers are trapped and released over time, the current gradually rises during illumination and then slowly declines back to its initial state after the light source is removed, as the mechanism depicted in Figure 3c.

The variation in electrical conductivity over time mimics synaptic plasticity, such as short‐term plasticity (STP), long‐term plasticity (LTP), and paired‐pulse facilitation (PPF), enabling functions related to learning and memory in neuromorphic computing systems under light.^[^ ^105^, ^106^ ^]^ These optical synaptic devices offer benefits like parallel processing, high‐speed operation, and potential compatibility with current optical communication infrastructure, addressing the drawbacks of traditional optoelectronic devices.^[^ ^101^, ^107^ ^]^ Despite the early stage of defect‐induced optical synaptic devices, further research into diverse material systems, defect engineering techniques, and device architectures is crucial for practical implementation in neuromorphic computing.

#### 2.2.5 Optoelectronic Memory Devices

Floating‐gate optoelectronic memory devices represent a crucial form of multifunctional photodetection technology, merging optoelectronic features with floating‐gate structures.^[^ ^108^, ^109^ ^]^ These carriers are stored and retrieved by the light illumination and electrical processes, providing potential benefits in non‐volatility, scalability, and data retention.^[^ ^110^, ^111^ ^]^ In Figure 3d, the floating‐gate transistor structure features a conductive floating gate isolated by a dielectric layer. In n‐type semiconductors, a high positive gate voltage accumulates a significant number of electrons in the channel, enabling carriers to traverse the dielectric layer as the tunnel layer to the floating gate.^[^ ^111^, ^112^ ^]^ Electrons stored in the floating gate influence the enhanced holes in semiconductors without external gate bias, leading to a low current memory state. Reverting the floating‐gate device to its initial state involves using light pulses to neutralize the effect of the negative gate bias on the semiconductor channel by combining photogenerated holes with stored electrons in the floating gate.

This integration of optics and memory offers opportunities for rapid and effective data storage and retrieval, with promising implications for optical data processing, image recognition, and neuromorphic computing.^[^ ^113^, ^114^ ^]^ However, the intricate device structure and integration challenges associated with floating‐gate optoelectronic memory devices impede practical deployment and application advancement. Overcoming hurdles related to enhancing performance, reliability, and scalability is crucial to facilitate broader acceptance and commercial viability of these technologies.

## 3 Low‐Dimensional Materials for Next‐generation Image Sensors

Low‐dimensional materials utilized in image sensors include 0D QDs, 1D nanowires/nanorods, 2D materials, and hybrid materials. The specific preparation methods for these materials and their corresponding image sensors are extensively discussed. Furthermore, an analysis is conducted to assess the advantages and disadvantages of each type of low‐dimensional material for their application in image sensors.

### 3.1 0D quantum Dots (QDs)

The QDs as representative 0D materials, have unique structures with exciton Bohr radius smaller than their size (1–20 nm), inducing the quantum confinement effect. Therefore, high optical absorption, tunable bandgaps, properties dependent on ligands, and cost‐effective solution processing techniques are achieved for the QDs, exhibiting wide applications in optoelectronics, the quantum confinement, and spintronics.^[^ ^115^ ^]^ The hot‐injection method is widely employed for synthesizing QDs with uniform size distribution and high quality. This method involves injecting nonmetallic precursors into a solution of metal precursors at elevated temperatures, resulting in the formation of QDs with excellent properties.^[^ ^116^ ^]^ By adjusting the ratio of lead precursors to sulfur precursors as well as the size, shape, defect in synthesized QDs, researchers have successfully achieved PbS, ZnS, CdS, and perovskite QDs with tunable energy bands and controllable properties.^[^ ^117^ ^]^

**Figure 4:**
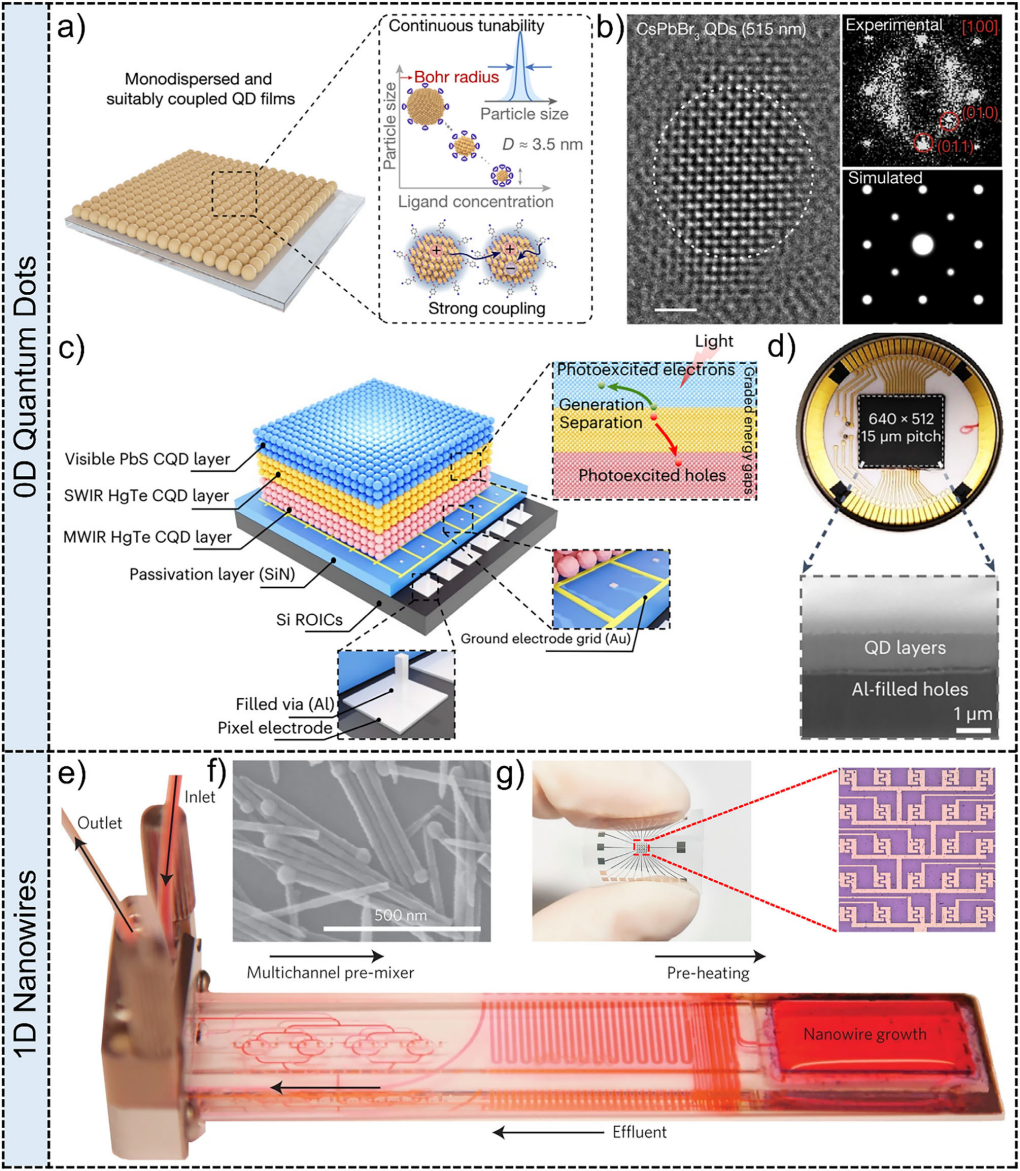
Quantum dots (0D) and nanowires (1D) for image sensors. quantum dots: a) Schematic diagram of the monodispersed and suitably coupled perovskite QD films prepared via single‐step spin‐coating. b) The HRTEM image of CsPbBr_3_ QDs. The right images are the corresponding FFT (fast Fourier transform pattern) image along^[^ ^100^ ^]^ direction and the SAED pattern. Scale bar: 2 nm. Reproduced with permission.^[^ ^118^ ^]^ Copyright 2022, Springer Nature Limited. c) Schematic diagram of the ultrabroadband imager consisting of PdS/HgTe CQDs and silicon circuits. d) The above image is the photograph of the ultrabroadband imager and the corresponding cross‐sectional SEM image. Reproduced with permission.^[^ ^122^ ^]^ Copyright 2024, Springer Nature Limited. 1D nanowires: e) The microfluidic reactor for synthesizing nanowires by a solution‐liquid‐solid (SLS) method. f) SEM image of synthesized CdSe nanowires. Reproduced with permission.^[^ ^126^ ^]^ Copyright 2013, Springer Nature Limited. g) Photograph of the flexible image sensor consisting of ordered S‐GaSb nanowires arrays with high sensitivity and optical images of the 5 × 5 pixels. Reproduced with permission.^[^ ^127^ ^]^ Copyright 2022, American Chemical Society.

**Figure** **4**a shows the schematic diagram of monodispersed and suitably coupled monodispersed perovskite QDs with ultrasmall‐size films by a direct synthesis‐on‐substrate method.^[^ ^118^ ^]^ By utilizing the α‐methyl‐benzyl‐ammonium (MBA^+^) with an extra methyl substitution (‐CH_3_) as the head group, steric hindrance is induced to suppresses the formation of layered perovskites. And the ligand concentration‐dependent particle size realizes the continuous tunability of the Bohr radius, resulting in the controllable PL and absorption spectra. Figure 4b is the low‐dose high‐resolution transmission electron microscope (HRTEM) image and the corresponding fast Fourier transform pattern of the synthesized QDs. The experimental results are consistent with the cubic phase of CsPbBr_3_ along the^[^ ^100^ ^]^ direction, which agrees well with the simulated results in right‐bottom image of Figure 4b.

QDs films have gained extensive applications in infrared imaging owing to their wide photoresponse range and high light absorption capabilities.^[^ ^119^, ^120^ ^]^ And the chemical solution methods for fabricating the QDs films offer compatibility with CMOS technology.^[^ ^20^, ^41^, ^121^ ^]^ Figure 4e is the schematic diagram of an ultra‐broadband focal plane array image sensor, comprising 640 × 512  pixels with three stacked layers of visible PbS CQDs, short‐wave infrared HgTe CQDs and mid‐wave infrared HgTe CQDs on a silicon readout integrated circuit.^[^ ^122^ ^]^ Figure 4d shows the photograph of the ultra‐broadband focal plane array image sensor and the corresponding cross‐sectional scanning electron microscopy (SEM) image. This PbS/HgTe CQDs photodetector has graded energy gaps and maximized vertical‐driven force, exhibiting ultra‐broadband spectral response from visible to mid‐wave infrared (0.4 to 5.0 µm) with excellent responsivity of 0.23, 0.31, 0.83 and 0.71 A·W^−1^ under illumination wavelength of 0.4, 0.7, 2.2 and 4.2 µm, respectively. Additionally, another PbS CQDs image sensor with 256 × 256  pixels demonstrates near‐infrared detection at 1.8 µm and has a *NEP* of 8.7 × 10^−11^ W at a frequency of 170 Hz.^[^ ^121^ ^]^ These image sensors utilizing QDs attract more and more attention in multispectral imaging and light intensity‐adaptive ultra‐broadband imaging.

### 3.2 1D Nanowires

1D inorganic nanostructures, such as carbon nanotubes (CNTs), silicon nanowires, ZnO nanowires, and perovskite nanowires, have diameters ranging from a few nanometers to several hundred nanometers, with length up to hundreds of microns.^[^ ^3^, ^89^ ^]^ The unique structure 1D nanowires lead to the special characteristic of enabling the transportation of charge carriers along a specific dimension while restricting movement in the other two dimensions.^[^ ^123^ ^]^ The excellent mechanical flexibility and the outstanding electrical and optoelectrical properties, have attracted great attention for strain sensors^[^ ^124^ ^]^ and integrated photodetectors for imaging.^[^ ^123^ ^]^ The near‐field electrospinning technique is widely used to construct nanowires and shows promise for depositing controllable large‐scale arrays of inorganic nanowires.^[^ ^125^ ^]^ By employing this technique, high‐performance ultraviolet detection has been achieved based on patterned Zn_2_GeO_4_ semiconductor nanowires arrays.^[^ ^22^ ^]^ Moreover, a blade coating method^[^ ^24^ ^]^ and a vapor‐solid‐solid reaction process^[^ ^23^ ^]^ are proposed to construct CH_3_NH_3_PbI_3_ nanowires arrays and 3D vertical CH_3_NH_3_PbI_3_ nanowires for image sensors.

To produce high‐quality, single‐crystalline nanowires, the solution‐liquid‐solid (SLS) method has been used by injecting chemical precursors into a hot surfactant solution and utilization of molten metal nanoparticles to catalyze the nucleation and growth of the nanowires.^[^ ^126^ ^]^ Figure 4e shows the diagram of an optimized SLS method utilizing a microfluidic reactor. This novel resealable microfluidic reactor allows for the controlled growth of nanowires from a solid substrate by flowing reactants and ligands over nanoparticle catalysts while efficiently removing reaction by‐products. The CdSe and ZnSe nanowires have been achieved by this method, where the diameter of nanowires is controlled by adjusting the size of catalyst Bi droplets. Figure 4f shows the SEM image of the synthesized CdSe nanowires. The controllable size of Bi droplets allows the exploration about the influence of reaction conditions on nanowire morphology and clarification of the mechanistic processes involved in solution‐catalyzed growth. The controllable fabrication of semiconductor nanowires lays the foundation for device integration and use in image sensors. Additionally, S‐GaSb nanowires with polarized photoresponse performances are integrated into a 5 × 5 devices array for flexible polarimetric image sensors.^[^ ^127^ ^]^ Figure 4g shows the photograph of the corresponding flexible polarimetric image sensor. This image sensor possesses polarization imaging capability under linear polarization 1.55 µm light. These results exhibit the great potential of 1D nanowires in integrated devices with superior imaging ability.

### 3.3 2D Materials

2D materials like 2D silicon, graphene, TMDs, and perovskite offer tunable optical bandgaps, unique optical/electrical properties, and mechanical flexibility, making them highly attractive for image sensor applications.^[^ ^67^, ^128^ ^]^ This section offers a summary of preparation methods, device structures, and the associated image sensors based on 2D silicon ribbons, TMDs, and perovskites.

#### 3.3.1 2D Silicon Ribbons

Supported by extensive research, mature integration technology, and cost‐effectiveness, silicon plays a prominent role in digital image systems.^[^ ^129^ ^]^ Stretchable form of silicon consisting of sub micrometer single‐crystal ribbons has been successfully constructed on elastomeric substrates for flexible image sensors.^[^ ^130^, ^131^ ^]^ Photolithography is applied to define patterned mask of a silicon‐on‐insulator wafer, which is then etched to eliminate the exposed areas of the silicon layer. After removing the resist using acetone, the patterned silicon ribbons are formed, which are released from substrated by etching the buried SiO_2_ layer with concentrated hydrofluoric acid. These silicon ribbons are lifted onto stretched poly(dimethylsiloxane) (PDMS) substrates, forming well‐defined waves‐like patterns. The stretchable p‐n junction diodes have electrical responses at various strain levels and maintain consistent electrical properties when subjected to stretching or compression. Additionally, a scalable and foldable integrated circuit, utilizing twisted and bent wavy monocrystalline silicon nanoribbons, has been successfully developed.^[^ ^132^ ^]^ These advancements address the challenge of 2D silicon deformation and establish a solid foundation for the application of silicon devices in flexible image sensors.

**Figure 5:**
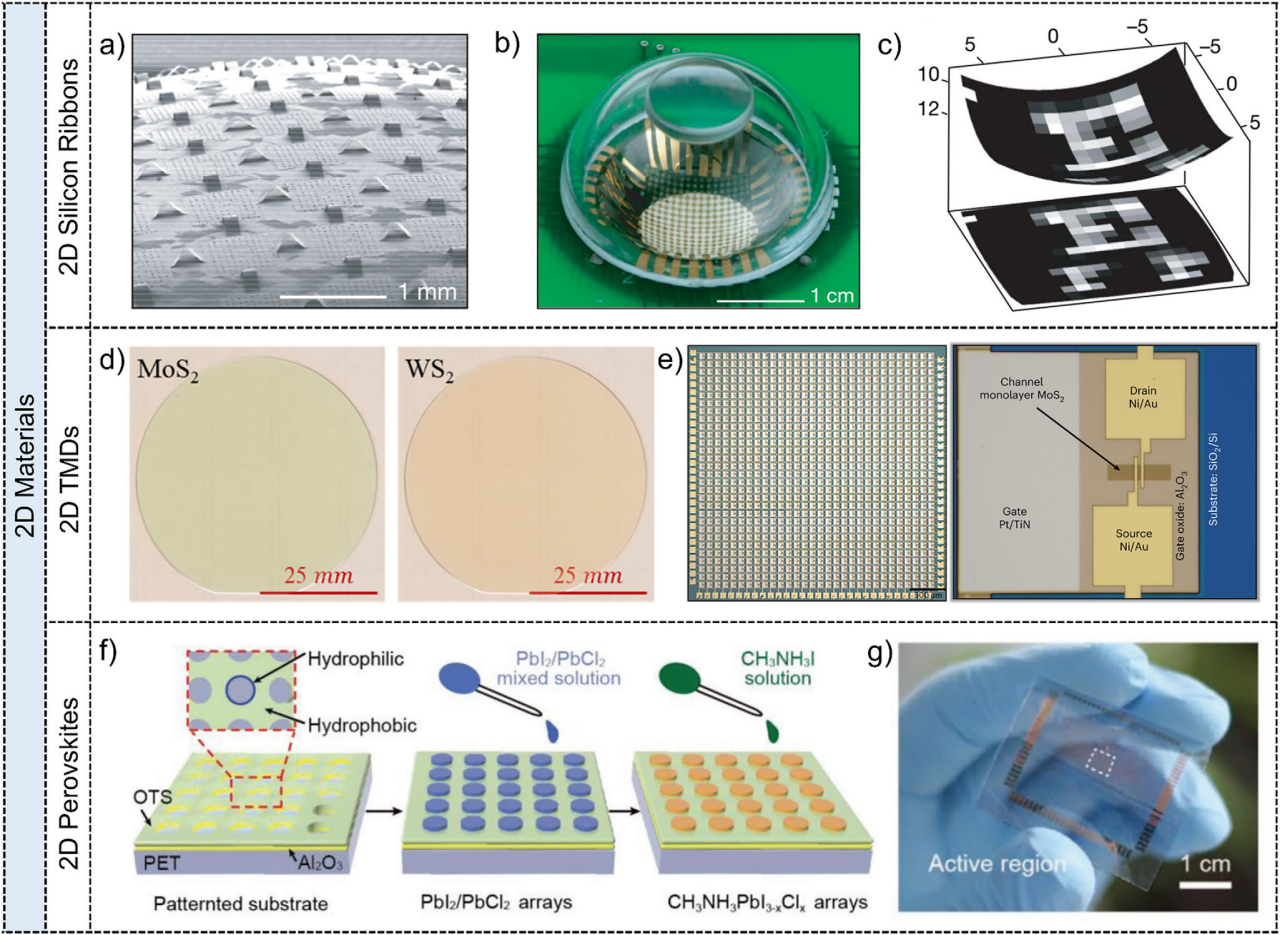
2D materials for image sensors. **2D silicon ribbons**: a) Photograph of the hemispherical electronic eye camera based on compressible silicon optoelectronics. b) SEM image of the silicon optoelectronic array on a PDMS transfer element. c) Greyscale images of the first two rows in an eye chart acquired using a hemispherical camera with a 16‐by‐16‐pixel array. Reproduced with permission.^[^ ^133^ ^]^ Copyright 2008, Springer Nature Limited. **2D TMDs**: d) MOCVD grown MoS_2_ and WS_2_ films on 2‐inch sapphire wafer. Reproduced with permission.^[^ ^143^ ^]^ Copyright 2021, Springer Nature Limited. e) Optical images of the image sensor consisting of 900 active pixels and a single pixel based on the monolayer MoS_2_ phototransistor. Reproduced with permission.^[^ ^144^ ^]^ Copyright 2022, Springer Nature Limited. **2D perovskites**: f) Synthesis process of patterned perovskite arrays by a deposition method. g) Optical image of a flexible perovskite photodetectors array consisting of 10 × 10 pixels. Reproduced with permission.^[^ ^147^ ^]^ Copyright 2018, WILEY‐VCH Verlag GmbH.

**Figure** **5**a displays a photograph of a hemispherical camera for curvilinear optoelectronics and electronic eye imagers, which consists of an array of curved silicon devices, a transparent hemispherical cap, and a basic imaging lens.^[^ ^133^ ^]^ The hemispherical PDMS flexible substrate is stretched at various angles to achieve a planar shape, and subsequently, the single‐crystalline silicon device array is transferred onto the planar PDMS to create the hemispherical silicon device array. Figure 5b illustrates the SEM diagram of the curved silicon device array, clearly depicting the bending of the monocrystalline silicon. Figure 5c displays the imaging result obtained from the hemispherical electronic eye camera featuring a 16 × 16‐pixel array. The clear identification of the resulting images demonstrates the remarkable imaging capabilities of the hemispherical image sensor. These findings underscore the exceptional performance and valuable applications of 2D single‐crystalline silicon in curved image sensors.

#### 3.3.2 2D Layered Materials

The discovery of graphene opens up the way for the research of 2D layered materials with van der Waals (vdWs) forces in adjacent layers and covalent bonding between atoms in each layer.^[^ ^134^, ^135^ ^]^ The remarkable electronic properties, ultra‐thin thickness, atomic flatness, and adjustable optoelectronic performance hold great promise for the advancement of future image sensors.^[^ ^12^, ^67^, ^136^, ^137^ ^]^ CVD and metal‐organic CVD (MOCVD) are bottom‐up growth methods, providing high‐quality growth and capability for scalable production for 2D layered materials. The CVD method usually employs solid precursors at high temperatures (700–1000 °C) in a horizontal tube furnace to synthesize large‐area and single‐crystalline TMDs. However, this method struggles with precise stoichiometric control with sulfur vacancies and requires temperature‐resistant substrates. In contrast, MOCVD utilizes volatile metal‐organic precursors and chalcogen hydrides at lower temperatures (400–600 °C), realizing superior stoichiometric accuracy and uniformity of TMDs. The MOCVD technique shows potential in industrial scalability and low‐temperature processing. However, the MOCVD process relies on costly and toxic precursors, coupled with potential carbon impurities from organic residues, limiting cost‐effectiveness and material purity.^[^ ^138^, ^139^, ^140^, ^141^, ^142^ ^]^ Uniform films of MoS_2_ and WS_2_ on 2‐inch sapphire wafers are successfully obtained by utilizing the MOCVD system, as shown in Figure 5d.^[^ ^143^ ^]^ A 900‐pixel 2D active pixel sensor (APS) matrix featuring monolayer MoS_2_ phototransistors has been fabricated, with individual phototransistors integrating programmable TiN/Pt/Al_2_O_3_ back‐gate islands as shown in Figure 5e.^[^ ^144^ ^]^ The photogate effect in MoS_2_, induced by structural defects and localized trap states at the MoS_2_/dielectric interface, allows for gate‐tunable photocarrier trapping. By applying large positive reset voltage pulses to the local back‐gate of the MoS_2_ phototransistor, efficient de‐trapping can be achieved, enabling effective de‐noising in 2D APS technology without the need for extensive peripheral circuitry or complex algorithms. Imaging functions utilizing 2D materials exhibit efficient noise‐reduction capabilities, showcasing the potential for next‐generation image sensors.

#### 3.3.3 Perovskite Films

Metal halide perovskites have special crystal structures described by a formula ABX_3_, where A, B and X denote a monovalent organic or inorganic cation (e.g., CH_3_NH_3_^+^, HC(NH_2_)_2_^+^, Cs^+^ or Rb^+^), a divalent metal cation (e.g., Pb^2+^, Sn^2+^, Cu^2+^ or Ge^2+^), and X represents a monovalent halide anion (e.g., Cl^−^, Br^−^ or I^−^), respectively.^[^ ^128^, ^145^ ^]^ Perovskites gain attention for their tunable bandgap and exceptional photoelectric properties in optoelectronic devices and solar cells.^[^ ^146^ ^]^ An effective two‐step sequential deposition method has been proposed to synthesize large‐scale CH_3_NH_3_PbI_3−x_Cl_x_ films.^[^ ^147^ ^]^ It should be noted that although CH_3_NH_3_PbI_3−x_Cl_x_ is normally considered as the 3D crystal, it is broadly included as a subset as 2D materials in this review because the x and y dimensions of the perovskite films are significantly larger than the z dimension. Figure 5f shows the schematic diagram about the process flow. The influence of hydrophilic‐hydrophobic surfaces on patterned perovskite films is crucial, particularly with the formation of an auxiliary patterned Al_2_O_3_ film on a flexible polyethylene terephthalate (PET) substrate. The Al_2_O_3_ film can be rendered hydrophobic using a mixed solution of hexane and octadecyltrichlorosilane, which is then patterned through photolithography and etching. Following this, oxygen plasma treatment makes the exposed areas of the substrate hydrophilic, allowing for the spin‐casting of a precursor solution containing PbI_2_ and PbCl_2_ in N,N‐dimethylformamide. Subsequently, CH_3_NH_3_PbI_3−x_Cl_x_ arrays are synthesized by spinning CH_3_NH_3_I solutions on the patterned precursor arrays. The crystallization quality of the resulting perovskite arrays is further improved by annealing at 100 °C for 30 min. The digital image in Figure 5g showcases a flexible perovskite photodetector array with 10 × 10 pixels with CH_3_NH_3_PbI_3−x_Cl_x_ as the light‐sensitive material and Ni/Au arrays as electrodes on flexible PET substrates, highlighting its exceptional flexibility and imaging capabilities. This research underscores the potential of 2D perovskites in photodetectors, imaging functionalities, and flexible optoelectronics, paving the way for practical applications in the future.

### 3.4 Hybrid Materials

Hybrid materials, blending diverse material traits, have been explored for next‐generation image sensors. Here we present three typical hybrid material categories: 0D and 1D composites, 0D and 2D composites, and 2D heterostructures, delving into their material compositions, device structures, and photoelectric properties in relation to image sensors.

#### 3.4.1 0D and 1D Composites

**Figure 6:**
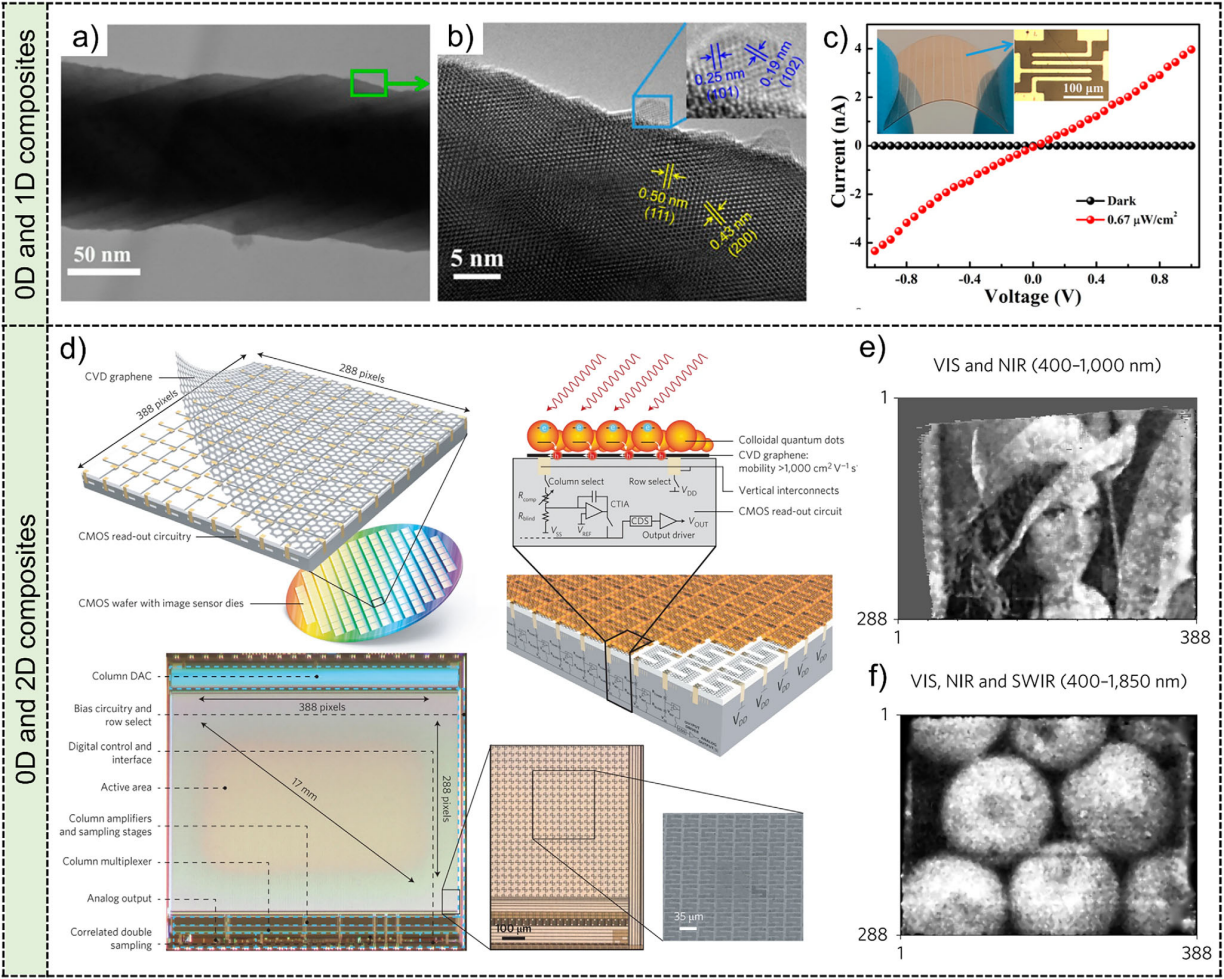
Low‐dimensional material composites for image sensors. **0D and 1D composites**: a) TEM image and b) HRTEM image of Zn_2_SnO_4_ nanowire with ZnO QDs. c) *I*−*V* curves of the photodetector based on Zn_2_SnO_4_ nanowire with ZnO QDs under dark and 300 nm UV illumination. Insets are the optical images of photodetectors on a flexible PET substrate. Reproduced with permission.^[^ ^151^ ^]^ Copyright 2017, American Chemical Society. **0D and 2D composites**: d) Schematic diagram of the process about transferring graphene film onto the CMOS circuitry. Schematic diagram of graphene‐CMOS image sensors. And photograph of the image sensor with ∼110000 photoconductive graphene channels without depositing CQDs. Photographs taken by the graphene‐CMOS image sensor deposited by CQDs e) with an exciton peak at 920 nm and f) 1670 nm. Reproduced with permission.^[^ ^20^ ^]^ Copyright 2017, Springer Nature Limited.

The 0D and 1D composites capitalize on the strengths of individual components, demonstrating superior light absorption capabilities and alluring properties through the strategic design of energy bands and interface structures within the devices.^[^ ^148^, ^149^, ^150^ ^]^ The ZnO QDs decorated Zn_2_SnO_4_ nanowires have been proposed as photosensitive materials in image sensors.^[^ ^151^ ^]^ The single‐crystalline Zn_2_SnO_4_ nanowires synthesized by CVD methods are subjected to solvothermal conditions in an autoclave for the growth of ZnO QDs. **Figure** **6**a,b shows transmission electron microscope (TEM) images, showing that the diameter of ZnO QDs is ≈10 nm on the Zn_2_SnO_4_ nanowire surface. The heterostructure interface traps holes in ZnO, elevating the electrostatic potential in the QDs and triggering a positive gating effect to boost the photoresponse. In Figure 6c, the output curves are displayed under dark conditions and UV illumination, exhibiting enhanced current under light illumination. The insets showcase optical images of a 10 × 10 photodetector array and a single device.

Moreover, other 0D and 1D hybrid nanostructures of SnS QDs/Zn_2_SnO_4_ nanowires have been fabricated using a two‐step CVD process.^[^ ^152^ ^]^ The photodetector exhibits excellent photoresponse ranging from 400 to 950 nm stems from the narrow bandgap (≈1.3 eV) of SnS QDs. And the high responsivity is a result of the intrinsic electric field at the SnS‐Zn_2_SnO_4_ interface, which leads to hole trapping in SnS QDs, suppresses electron‐hole pair recombination, and enhances broadband photoresponse. Furthermore, A broadband image sensor array utilizing SnS QD‐coated Zn_2_SnO_4_ nanowires, is constructed on a flexible PET substrate. The system captures clear images of a smiling face and a bird under white and red light, showcasing effective target identification. These sensors demonstrate versatile UV to NIR imaging capabilities, highlighting the potential of 0D and 1D composites in flexible imaging technologies.

#### 3.4.2 0D and 2D Composites

The integration of 0D quantum dots with 2D materials to boost light absorption and enhance photodetection sensitivities have been extensively explored.^[^ ^67^, ^153^, ^154^, ^155^ ^]^ This integration has been observed in various technologies, such as MoS_2_‐PbS quantum dot photodetectors,^[^ ^156^ ^]^ graphene‐CsPbBr_3_ QDs optoelectronic synapses,^[^ ^157^ ^]^ and upconverting nanoparticles‐MoS_2_ floating gate phototransistor. In these setups, 2D materials typically serve as light‐sensitive semiconductor channels, and 0D QDs as light absorption layer.

Figure 6d shows an image sensor with a 388 × 288 array consisting of graphene‐CQD photodetectors.^[^ ^20^ ^]^ Each pixel in the image sensor includes a PbS CQD layer, a graphene layer and the bottom CMOS. Photogenerated carriers in the PbS CQD layer are transferred into the graphene layer to enhance the conductance. An ultrahigh gain of 10^8^, responsivity above 10^7^ A·W^−1^ and a detectivity exceeding 10^12^ cm·Hz ^1/2^W^−1^ are achieved. The bottom image in Figure 6d shows the photograph of the image sensor with ≈110 000 photoconductive graphene channels. The scanning electron micrograph (SEM) of the active area displays an S‐shaped pixel structure. Integrating the graphene‐CQD image sensor with CMOS circuitry enables impressive imaging capabilities. Figure 6e,f exhibits images captured by the graphene‐CQD sensor with exciton peaks at 920 and 1670 nm respectively. These outcomes underscore the sensor's sensitivity across ultraviolet, visible, and infrared light (300–2000 nm), highlighting the vast potential of 0D‐2D hybrid materials in advanced broadband and high‐resolution imaging systems.

#### 3.4.3 2D–2D Junctions

**Figure 7:**
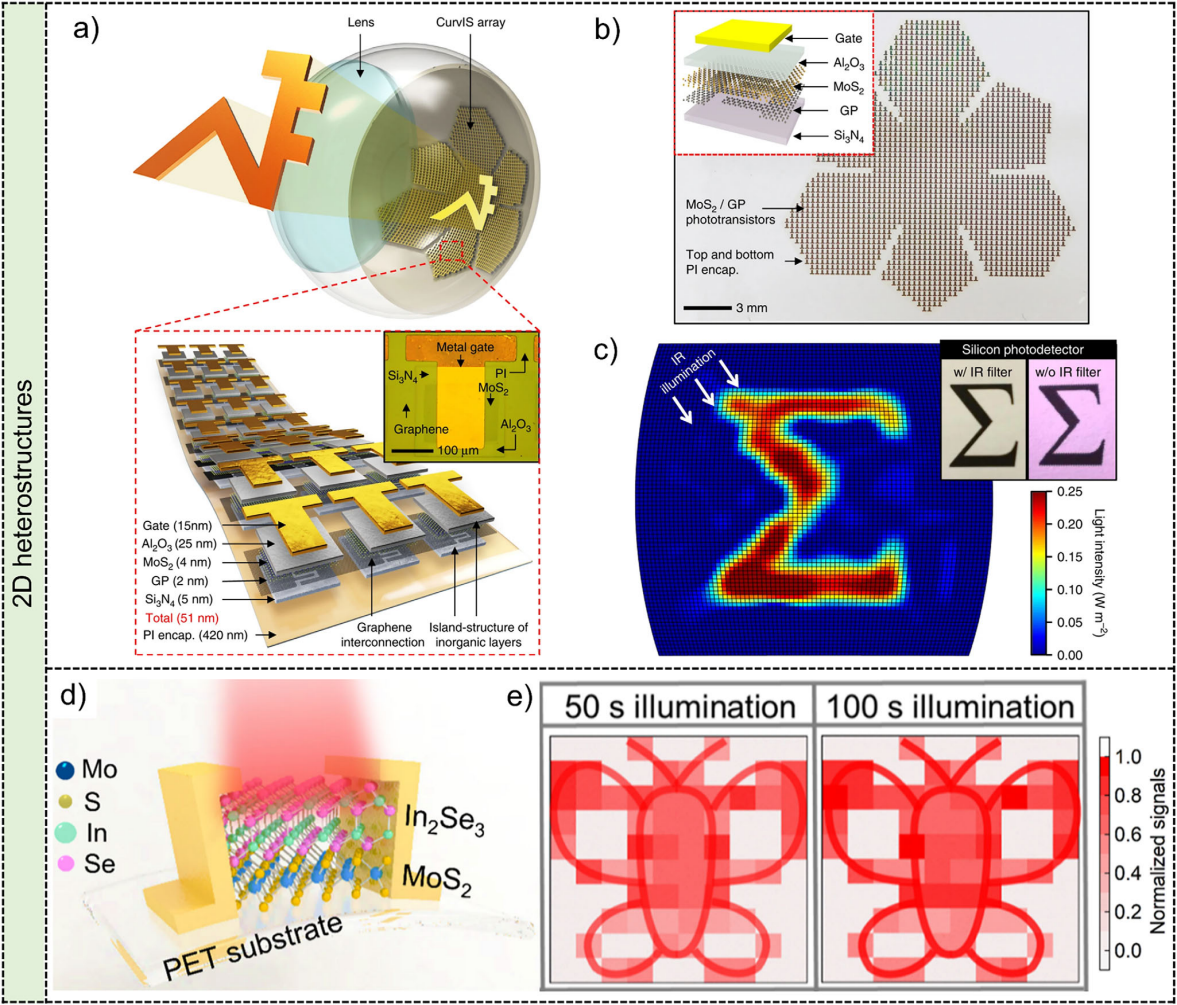
2D heterostructures for image sensors. a) Schematic illustration of the high‐density curved image sensor array based on the MoS_2_/graphene heterostructure. b) The optical camera image of the designed phototransistor array on a planar substrate. Inset is the schematic illustration of a single device structure. c) Sigma pattern captured by the curved image sensor array. Reproduced with permission.^[^ ^159^ ^]^ Copyright 2017, Springer Nature Limited. d) Schematic of the In_2_Se_3_/MoS_2_ heterostructure device on the flexible PET substrate. e) Butterfly image attained by the In_2_Se_3_/MoS_2_ heterostructure devices array under 1060 nm illumination for 50 s and 100 s. Reproduced with permission.^[^ ^44^ ^]^ Copyright 2022, American Chemical Society.

The tunable energy bands and broad optical absorption capabilities of 2D materials with layered structures sparked significant interest in image sensors. Heterostructures composed of multiple 2D materials present advantages through tailored component and device designs, promising applications in image sensors.^[^ ^158^ ^]^ **Figure** **7**a shows the schematic diagram and digital image of high‐density curved image sensors based on ultrathin MoS_2_‐graphene heterostructures.^[^ ^159^ ^]^ The array design features a truncated icosahedron layout to prevent folds and wrinkles. Each device comprises a phototransistor with MoS_2_‐graphene heterostructures as channels, Al_2_O_3_ as the dielectric, and Ti/Au as the electrodes. The phototransistor array with special structures design is first constructed on a flat substrate with the polyimide encapsulation (Figure 7b), then transferred to a hemispherical surface to fabricate the curved image sensors. Inset image in Figure 7b is the detailed device structure of each phototransistor. Patterned images under infrared radiation are acquired by curved image sensors employing MoS_2_‐graphene heterostructures, showcased in Figure 7c. This work demonstrates advantages of 2D heterostructures in curved image sensors, a step forward to the hemispherical retinal prosthesis and next‐generation flexible electronics.

The interfaces within the heterostructures of 2D layered materials are tailored to enable synaptic capabilities.^[^ ^153^, ^160^, ^161^, ^162^ ^]^ A In_2_Se_3_/MoS_2_ heterostructure is constructed into a two‐terminal device on a flexible PET substrate, demonstrating essential synaptic functions under NIR illumination.^[^ ^44^ ^]^ Figure 7d illustrates the flexible In_2_Se_3_/MoS_2_ synaptic device. By forming the heterostructure, the bandgap is reduced, enabling a photoresponse under NIR light. The potential barriers at the In_2_Se_3_/MoS_2_ interface impede carrier recombination, facilitating optical synaptic plasticity. The 10 × 10 In_2_Se_3_/MoS_2_ synaptic devices array in Figure 7e shows imaging functions under NIR illumination. The “butterfly” pattern becomes more discernible with extended illumination, correlating with increased currents. This study highlights the utility of 2D heterostructures in flexible synaptic devices and NIR image sensors, paving the way for innovative research in intelligent image sensors and target recognition.

## 4 Applications for Image Sensors

Traditional visible image sensors are based on silicon‐based CMOS and infrared image sensors are based on indium gallium arsenide (InGaAs) or mercury cadmium telluride (HgCdTe), showing wide application value in digital imaging, night monitoring, meteorology and military.^[^ ^41^, ^163^, ^164^, ^165^ ^]^ Nevertheless, the limitations imposed by the 3D structure of materials hinder the progress of image sensor technology. Thus, image sensors employing low‐dimensional materials with unique atomic structures, exceptional flexibility, superior optoelectronic properties, and compatibility with CMOS processes hold significant promises for advanced imaging systems, biomimetic vision sensors, and non von Neumann computing architectures.

The structure‐performance relationships of low‐dimensional semiconductor materials in photodetection performance largely determines the application fields of next‐generation image sensors. For advanced imaging sensors, 0D materials (e.g., PbS^[^ ^20^ ^]^ and HgTe^[^ ^122^ ^]^ QDs) with broad spectral range, tunable bandgaps, and high light absorption, are suitble for the image sensors with high performance yet face challenges in stability and quantum efficiency under prolonged operation. Moreover, 1D materials (e.g., Bi_2_Se_2_S^[^ ^50^ ^]^ nanowires), 2D materials (e.g., MoS)_2_^[^ ^97^ ^]^ and corresponding junctions (e.g., 2H‐MoTe_2_ homojunctions)^[^ ^166^ ^]^ offer exceptional carrier mobility and ultrathin flexibility, showing potentials in image sensors with high performance and self‐powered image sensors, but struggle with scalable integration and dark current noise. Moreover, the mechanical flexibility of 1D nanowires^[^ ^45^ ^]^ and 2D films^[^ ^46^ ^]^ facilitates the construction of flexible and hemispherical device arrays, making them excellent candidates for biomimetic vision sensors. However, there is still a lack of a universal and mature method for preparing large‐scale uniform low‐dimensional semiconductor materials on flexible or hemispherical substrates. Currently, non von Neumann imaging systems exploit defect‐engineered low‐dimensional semiconductor materials^[^ ^167^ ^]^ and designed heterostructures^[^ ^53^ ^]^ for in‐sensor memory and neuromorphic computing, yet their intricate device architectures and interfacial defects pose challenges for uniformity and energy efficiency. Overall, the choice of low‐dimensional materials directly dictates performance trade‐offs: 0D materials prioritize spectral versatility, 1D or 2D materials emphasize mechanical adaptability, and hybrid systems balance multifunctionality with integration hurdles. This section offers a systematic introduction of the performance metrics and application‐specific benefits of low‐dimensional semiconductor materials, along with their implementations in advanced imaging systems, biomimetic vision technologies, and non‐von Neumann computing architectures.

### 4.1 Advanced Image Sensors

**Figure 8:**
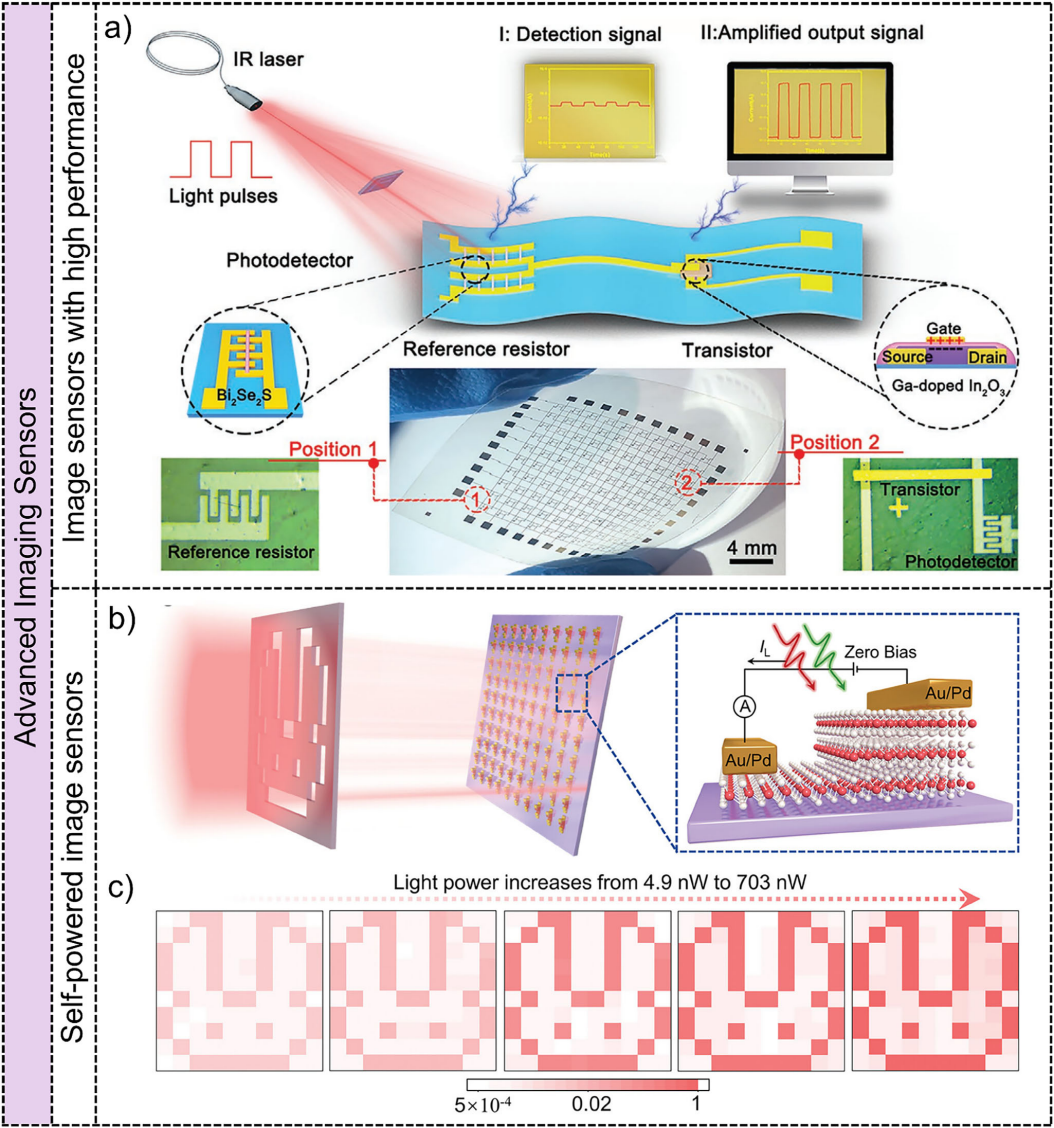
Advanced image sensors based on low‐dimensional materials. **Image sensors with high performance**: a) Schematic diagram of the infrared detection amplification (IRDA) system by integrating the device of Bi_2_Se_2_S nanowire‐based photodetector and Ga‐doped In_2_O_3_ nanowire based FET. Reproduced with permission.^[^ ^50^ ^]^ Copyright 2020, WILEY‐VCH Verlag GmbH. **Self‐powered image sensors**: b) The schematic diagram of the self‐powered imaging process via the image sensors consisting of 10 × 10 2H‐MoTe_2_ homojunctions with asymmetric thickness. c) The light power‐dependent imaging results under 1060 nm illumination without external bias. The contrast of image results increases with the enhancement of light power of 1060 nm light source. Reproduced with permission.^[^ ^166^ ^]^ Copyright 2020, WILEY‐VCH Verlag GmbH.

Advanced image sensors feature enhanced photoresponsivity, broad‐spectrum detection, polarimetric capabilities, and self‐powered operation, extending their application versatility. Augmenting photosensitivity is crucial for achieving high‐contrast, low‐noise imaging outcomes. Inspired by external amplification circuits that enhance processing efficiency and image recognition rates,^[^ ^42^, ^168^ ^]^ an infrared range detection amplification (IRDA) system based on low‐dimensional materials has been developed.^[^ ^50^ ^]^ The integrated IRDA system has been shown in **Figure** **8**a, which consists of a detector and a reference resistor based on single‐crystalline Bi_2_Se_2_S nanowires, and a Ga‐doped In_2_O_3_‐based top‐gate transistor. Under illumination state, the photodetector has lower resistance than that at dark conditions, which induces a small negative gate voltage of the top‐gate transistor, showing an On‐state of the IRDA system with high on/off ratio and photocurrent. The IRDA system significantly enhances photosensitivity to 7.6 × 10^4^, several orders of magnitude greater than that of the Bi_2_Se_2_S nanowire photodetector. Additionally, a 10 × 10‐pixel IRDA array has been created, effectively demonstrating imaging capabilities under patterned light. The enhanced photoresponsivity, combined with improved noise reduction and contrast, emphasizes the potential of IRDA system for advanced image sensors and encourages innovative designs for high photoresponsivity imaging devices.

Self‐powered photodetectors utilizing low‐dimensional materials through the photovoltaic effect have achieved by engineering an asymmetric depletion region within homojunction, heterojunction, and Schottky junctions.^[^ ^39^, ^169^ ^]^ Single‐pixel imaging systems consisting of mechanically exfoliated 2D heterostructures such as an InSe/WSe_2_/SnS_2_ heterojunction have been investigated, for self‐powered photodetector with enhanced built‐in electric fields.^[^ ^170^ ^]^ Recently, a multi‐pixel imaging system consisting of a patterned van der Waals homojunctions array based on 2H‐MoTe_2_ layers with asymmetric thickness has been proposed.^[^ ^166^ ^]^ Due to the energy bands of 2H‐MoTe_2_ layers as a function of thickness, the built‐in electric field is formed at the interface between thick and thin 2H‐MoTe_2_ layers and modulated by the thickness difference in the 2H‐MoTe_2_ homojunction. Figure 8b shows the schematic diagram of the imaging process through the 10 × 10 2H‐MoTe_2_ homojunctions array. The NIR light selectively illuminates the image sensor through a “rabbit” mask without external bias. The corresponding light power‐dependent imaging results are shown in Figure 8c. The shape and contrast of rabbit‐pattern images become more and more clear with the increase of the light power of NIR light, demonstrating the self‐powered imaging function and the modulation capability of light power. The construction and integration of pristine van der Waals homojunctions provides novel strategies for the development of 2D materials in advanced image sensors with ultralow power consumption and wide spectra range.

### 4.2 Biomimetic Vision Sensors

Biological eyes serve as the primary sensory organs for many animals, leading to the development of biomimetic devices that replicate the structure and functions of natural organisms.^[^ ^171^, ^172^, ^173^ ^]^ Most biomimetic vision systems are inspired by vertebrate and insect eyes, which are renowned for their exceptional optical capabilities, including high resolution, flexibility, and a wide field of view.^[^ ^174^, ^175^, ^176^ ^]^ Furthermore, these biomimetic vision sensors are engineered for minimally invasive diagnostics and high‐resolution biological imaging.^[^ ^5^ ^]^ For instance, wearable and implantable devices integrate biological sensors and electronic systems to monitor real‐time physiological changes,^[^ ^177^ ^]^ such as body temperature^[^ ^178^ ^]^ and blood flow dynamics.^[^ ^179^ ^]^ In endoscopy, ultrathin, conformable image sensors mimicking insect compound eyes could enable high‐resolution imaging of internal tissues, detecting early‐stage tumors or inflammation without invasive procedures.^[^ ^180^ ^]^ Inherently soft low‐dimensional semiconductor materials are promising candidates for photo‐absorbing components in high‐density flexible arrays due to their exceptional qualities, including high photo‐absorption coefficients, photoresponsivity, and significant fracture strain.^[^ ^181^, ^182^, ^183^ ^]^

**Figure 9:**
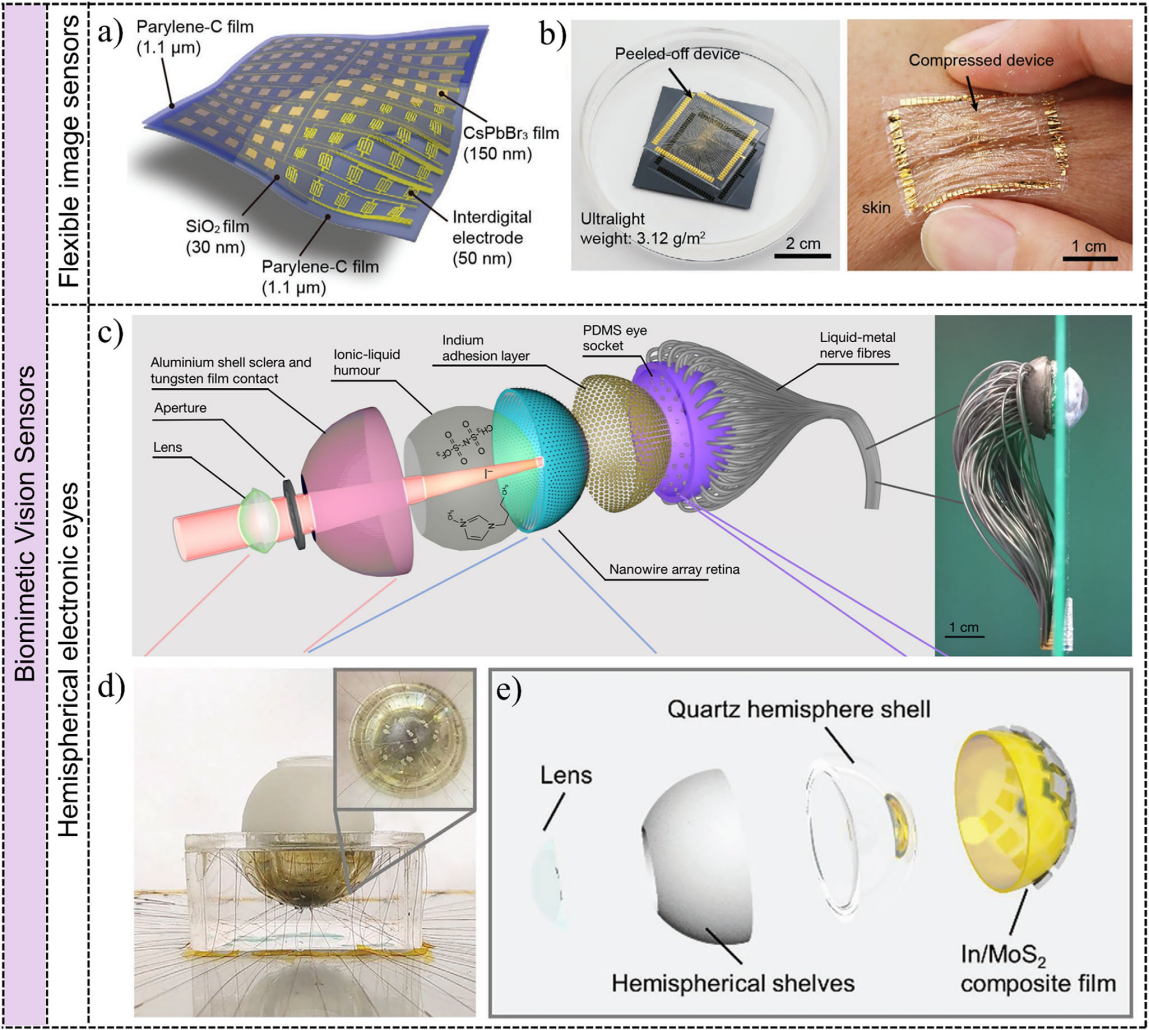
Biomimetic vision sensors based on low‐dimensional materials. **Flexible image sensors**: a) Schematic diagram of the flexible image device based on perovskite films, the cross‐section image of an individual pixel and the optical image of the photodetectors array. b) Digital image of the ultra‐lightweight device in NaCl solution, and the compressed device on the skin. Reproduced with permission.^[^ ^52^ ^]^ Copyright 2022, Elsevier Ltd. **Hemispherical electronic eyes**: c) Exploded view of the biomimetic electrochemical eye based on perovskite nanowire arrays. Right image is the photograph of the electrochemical eye. Reproduced with permission.^[^ ^45^ ^]^ Copyright 2020, Springer Nature Limited. d) Photograph of an electronic eye with a hemispherical In/MoS_2_ synaptic devices array. e) The corresponding detailed structure of the electronic eye. Reproduced with permission.^[^ ^46^ ^]^ Copyright 2021, WILEY‐VCH Verlag GmbH.

All‐inorganic perovskite materials with remarkable electrical and optoelectrical properties and low‐cost patterned growth method are one of the most promising materials for the application in flexible image sensors.^[^ ^3^, ^184^ ^]^ An ultrathin image sensor has been fabricated by applying stable all‐inorganic CsPbBr_3_ films and waterproof parylene‐C films.^[^ ^52^ ^]^ **Figure** **9**a illustrates the structure of an ultrathin 10 × 10 photodetector array, highlighting the design of a single photodetector. The floating devices in a NaCl solution shown in Figure 9b demonstrate the ultralight nature of the image sensor. And the right image in Figure 9b depicts the image sensor compressed on human skin, showcasing its remarkable mechanical properties and elastomer‐like flexibility without delamination. Additionally, a vdWs CH_3_NH_3_PbI_3_/graphene heterostructure on a flexible PET substrate has been proposed to enhance light‐substance interaction.^[^ ^185^ ^]^ A three‐color image is captured, demonstrating the color recognition capability. These advancements highlight the potential of flexible image sensors based on low‐dimensional materials in flexible imaging systems with advanced functionalities.

Furthermore, the concave hemispherical retina in human eyes is remarkable for its wide field of view (FOV) of 150°–160° and its ability to simplify optical systems by directly compensating for aberrations from the curved focal plane, inspiring the research interest in hemispherical electronic retinas consisting of low‐dimensional materials.^[^ ^133^, ^186^, ^187^ ^]^ A fractal web design for a hemispherical photodetector array incorporating organic‐dye‐sensitized graphene has been developed to enhance light‐absorbing capabilities.^[^ ^188^ ^]^ The 48‐pixel pyronin B‐doped graphene photodetectors array on a rigid silicon wafer are transferred to a transparent hemispherical dome, forming a hemispherical image sensor. The curved imaging functions are achieved by adjusting the position of laser beam and focusing on spot sizes. Furthermore, an electrochemical eye featuring a hemispherical retina constructed from a high‐density array of perovskite nanowires has been developed to mimic the photoreceptors of the human retina.^[^ ^45^ ^]^ Figure 9c illustrates the detailed structure and photograph of this electrochemical eye. Light focused by a convex lens stimulates the photosensitive perovskite nanowires array, causing current variations and curved imaging functions. This setup successfully captures the character “A” with 100 pixels, achieving FOV of 100.1°, which approaches the human eye's FOV of 130°. These innovative designs of artificial eyes offer promising advancements in curved imaging functions and biomimetic vision systems.

Moreover, researchers are increasingly focused on enhancing imaging performance including low power consumption and high photo response. A notable advancement is the development of a hemispherical electronic retina featuring a 5 × 5 array of indium (In)/MoS_2_ synaptic devices, which exhibits ultralow power consumption for imaging tasks.^[^ ^46^ ^]^ Figure 9d,e shows the photograph of the biomimetic eye and the corresponding detailed structure, respectively. Each pixel in this setup functions as an In/MoS_2_ optical synaptic device with a two‐terminal configuration. The work function of indium (4.1 eV) is lower than that of MoS_2_ (4.7 eV), allowing electrons to flow from indium to MoS_2_ until equilibrium is reached. This results in a downward bending of the conduction band in MoS_2_, leading to enhanced conductivity and an impressive power consumption of just 68.9 aJ per spike for the optical synapse. As illumination time increases, the clarity of the curved images improves, showcasing the hemispherical imaging capabilities of this electronic eye. This research paves the way for advancements in artificial vision systems, particularly in enhancing the synaptic properties and imaging functionalities of biomimetic devices.

### 4.3 Non von Neumann Imaging Systems

Commercial imaging systems utilize the traditional von Neumann architecture, which comprises photoreceptors for capturing visual information, a memory unit for data storage, and a central processing unit for complex data processing.^[^ ^110^, ^189^ ^]^ This separation of components, along with linear interactions and extensive data transmission between them, results in high power consumption, slow data transfer speeds, and redundant data. These limitations hinder the advancement of computing systems.^[^ ^190^ ^]^ To address these challenges, researchers are exploring non‐von Neumann architectures that integrate sensing, memory, and computation, which can revolutionize intelligent imaging systems in AI‐driven and Internet of Things (IoT) applications. Image sensors integrated with neural network computing facilitate real‐time facial recognition and anomaly detection directly at the edge, finding applications in smart security monitoring.^[^ ^191^ ^]^ Non‐von Neumann systems improve energy efficiency by combining adaptive imaging with decision‐making, such as adjusting room lighting based on occupant presence and ambient light, while also analyzing gestures for appliance control.^[^ ^192^ ^]^ Additionally, these sensors enable predictive maintenance by examining thermal or spectral patterns from machinery to detect faults before failures occur in industrial IoT.^[^ ^193^ ^]^ This non von Neumann imaging system is gaining increasing attention as a means to overcome the issues related to the von Neumann bottleneck, memory wall, and power consumption wall.^[^ ^54^, ^194^ ^]^

**Figure 10:**
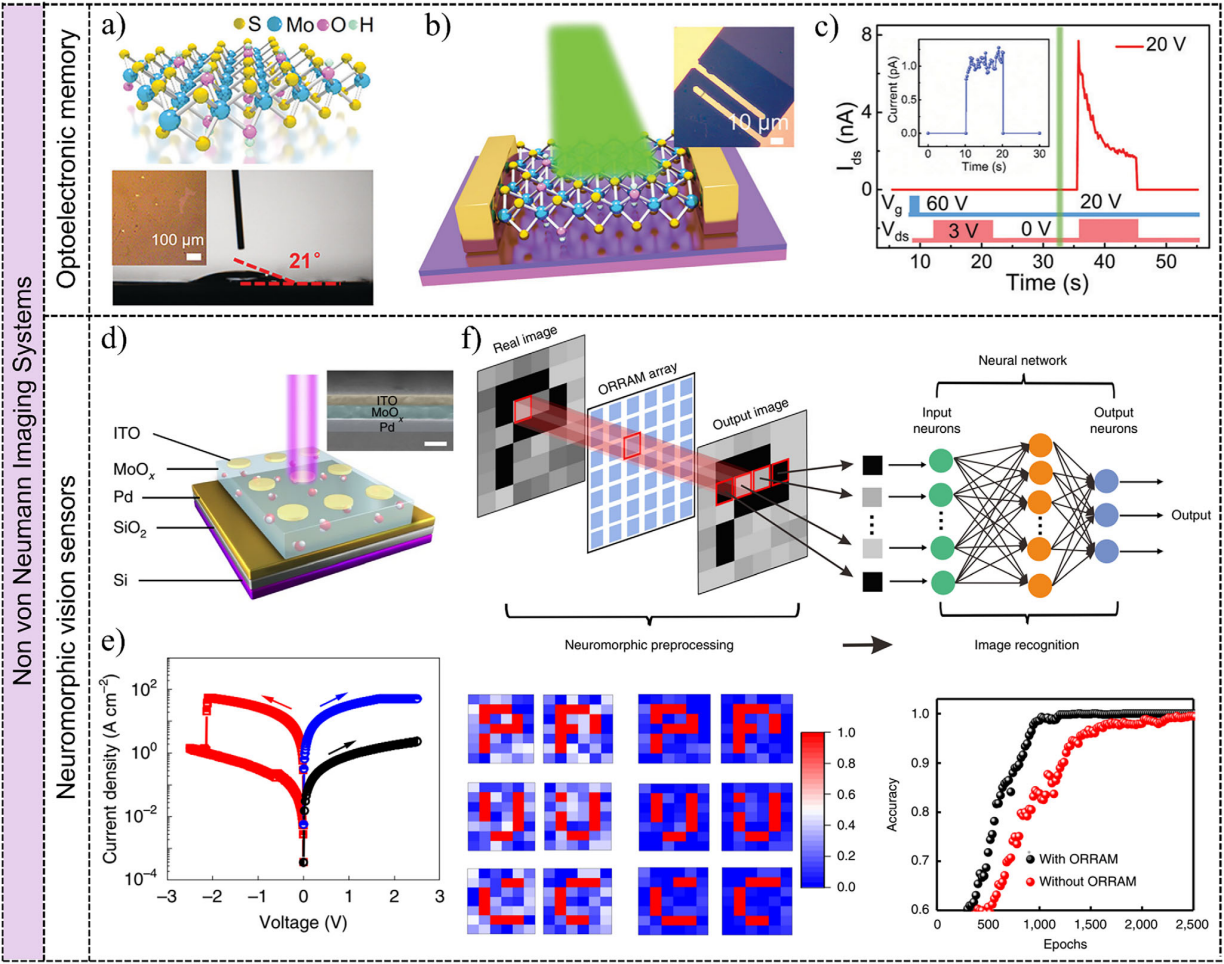
Non von Neumann imaging systems based on low‐dimensional materials. **Optoelectronic memory**: a) Schematic diagram and the contact angle of MoS_2_ with hydroxyl groups. b) Schematic of the optical memory transistor based on MoS_2_ under 550 nm illumination. Inset is the corresponding optical image of the device. c) An operating sequence of optical memory behavior based on the MoS_2_ transistor at 20 V gate voltage. Reproduced with permission.^[^ ^167^ ^]^ Copyright 2020, Royal Society of Chemistry. **Neuromorphic vision sensors**: d) Schematic diagram Schematic structure of the MoO_x_ ORRAM. e) Voltage sweeping based on the MoO_x_ ORRAM before and after UV illumination. f) Schematic of the neuromorphic visual system with the ORRAM‐based pre‐processing function, and the images contrast and recognition rate enhancement based on this neuromorphic visual system. Reproduced with permission.^[^ ^54^ ^]^ Copyright 2019, Springer Nature Limited.

The design and fabrication of optoelectronic memory devices utilizing low‐dimensional materials merge optical response with memory capabilities, demonstrating significant potential for neuromorphic visual systems to address the von Neumann bottleneck.^[^ ^195^, ^196^ ^]^ **Figure** **10**a shows a monolayer hydrophilic MoS_2_ film featuring covalently bonded hydroxyl groups that enhance its hydrophilicity by replacing sulfur atoms.^[^ ^167^ ^]^ The hydroxyl groups introduce traps within the MoS_2_ film, enabling optical memory functions and the development of optical memory transistors. The corresponding optical image and schematic illustration of the MoS_2_ optical memory transistor are shown in Figure 10b. Figure 10c shows one operation cycle of the nonvolatile characteristics and inset is the Off‐state current. By manufacturing the initial large gate pulse for low Off‐state current and light pulses for large On‐state current, optical memory functions and corresponding imaging results under different wavelengths after a 1‐min waiting period are obtained.

Moreover, the construction of heterostructures to modulate carrier behaviors through electricity and light is essential for realizing optical memory functions. A proposed heterostructure combining monolayer WSe_2_ and few‐layer hBN creates a multi‐bit nonvolatile optoelectronic memory device and an integrated pixel matrix with imaging capabilities.^[^ ^53^ ^]^ By applying gate and source‐drain voltages, the device perform programming, readout, and erasing processes. A 3 × 9 array of WSe_2_/hBN devices has been fabricated, demonstrating the ability to detect and differentiate light wavelengths using three laser beams (638, 515, and 473 nm), showcasing the potential for filter‐free color image sensors. These findings are crucial for advancing artificial visual systems based on non‐von Neumann architecture, potentially enhancing the development of practical applications such as cameras and fax machines.

Image learning significantly enhances image sensors by enabling automatic feature extraction, improving accuracy in object detection and classification, adapting to varying conditions, and facilitating real‐time processing, thereby transforming raw visual data into actionable insights for diverse applications.^[^ ^197^, ^198^ ^]^ Synaptic devices based on low‐dimensional materials exhibit increasingly broad application prospects in artificial machine vision.^[^ ^18^, ^199^ ^]^ A two‐terminal optoelectronic resistive random access memory (ORRAM) based on a structure of Pd/MoO_x_/ITO has been constructed with synaptic plasticity under optical stimulation.^[^ ^54^ ^]^ Figure 10d illustrates the schematic structure of the device, accompanied by a cross‐sectional SEM image. The electrical characterization in Figure 10e reveals that the device initially operates in a high resistance state (HRS) and can switch to a low resistance state (LRS) under a 365 nm laser. The LRS is maintained after light removal and reverts to its original state upon applying a voltage of −2.13 V. Therefore, ORRAM arrays achieve functions such as image sensing, memory, and contrast enhancement. Furthermore, simulations in Figure 10f utilize a three‐layer artificial neural network for image recognition, significantly improving the recognition rate.

Additionally, accurate environmental perception across varying illumination conditions is essential for horizontal cells and photoreceptors in the retina, capable of handling light intensity variations up to 280 dB. Advances in non‐von Neumann optoelectronic devices have shown promise for machine vision with visual adaptation. A bilayer MoS_2_ transistor with a high‐κ dielectric and charge trap states has achieved a dynamic range of 199 dB, significantly exceeding the 70 dB range of silicon CMOS, allowing for effective capture of both shadowed and highlighted image details.^[^ ^181^ ^]^ A three‐layer artificial neural network trained on the Modified National Institute of Standards and Technology (MNIST) dataset shows improved image recognition accuracy over time, with rates increasing significantly from 38.6% to 96.9% for scotopic adaptation and from 9.5% to 96.1% for photopic adaptation. These findings suggest that non‐von Neumann architectures utilizing low‐dimensional materials for image sensors, with their intensity and time‐dependent optoelectronic properties, hold substantial promise for bioinspired in‐sensor memory and adaptation functions, enhancing machine vision applications and simplifying hardware and algorithm complexities.

## 5 Summary and Outlook

**Figure 11:**
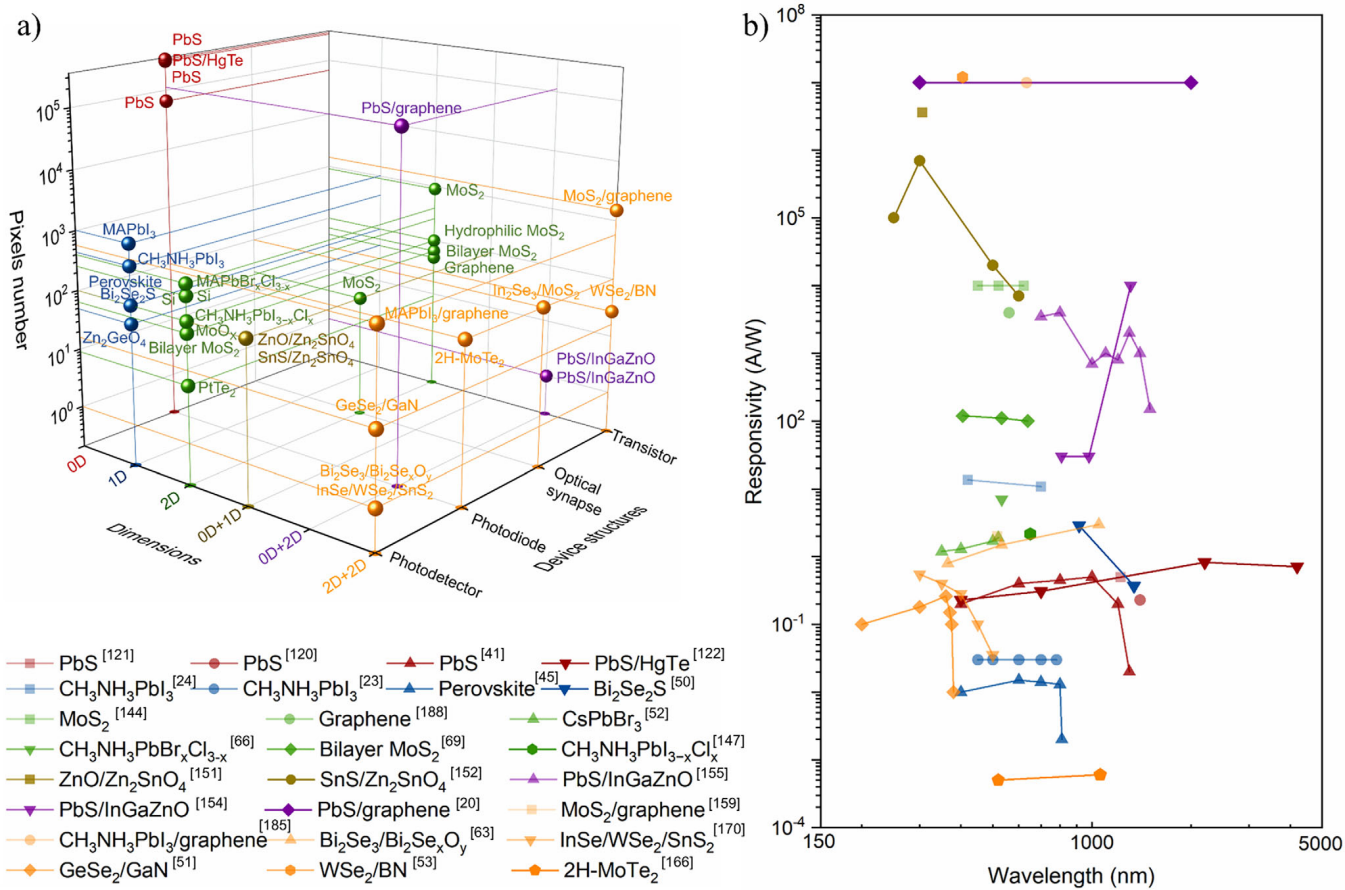
A summary of the reported image sensors based on low‐dimensional materials. a) A summary of pixel numbers in image sensors based on low‐dimensional materials (0D, 1D, 2D, 0D and 1D hybrids, 0D and 2D hybrids, 2D heterostructures) with diverse device structures (photodetectors, photodiodes, optical synapses, and transistors). b) A summary of the photoresponsivity and photodetection wavelength range of the single pixel with photodetectors, photodiodes, and transistors structures in the image sensors based on low‐dimensional materials, including PbS,^[^ ^41^, ^120^, ^121^ ^]^ PbS/HgTe,^[^ ^122^ ^]^ CH_3_NH_3_PbI_3_,^[^ ^24^ ^]^ CH_3_NH_3_PbI_3_,^[^ ^23^ ^]^ perovskite,^[^ ^45^ ^]^ Bi_2_Se_2_S,^[^ ^50^ ^]^ MoS_2_,^[^ ^144^ ^]^ graphene,^[^ ^188^ ^]^ CsPbBr_3_,^[^ ^52^ ^]^ CH_3_NH_3_PbBr_x_Cl_3‐x_,^[^ ^66^ ^]^ Bilayer MoS_2_,^[^ ^69^ ^]^ CH_3_NH_3_PbI_3−x_Cl_x_,^[^ ^147^ ^]^ ZnO/Zn_2_SnO_4_,^[^ ^151^ ^]^ SnS/Zn_2_SnO_4_,^[^ ^152^ ^]^ PbS/InGaZnO,^[^ ^154^, ^155^ ^]^ PbS/graphene,^[^ ^20^ ^]^ MoS_2_/graphene,^[^ ^159^ ^]^ CH_3_NH_3_PbI_3_/graphene,^[^ ^185^ ^]^ Bi_2_Se_3_/Bi_2_Se_x_O_y_,^[^ ^63^ ^]^ InSe/WSe_2_/SnS_2_,^[^ ^170^ ^]^ GeSe_2_/GaN,^[^ ^51^ ^]^ WSe_2_/BN,^[^ ^53^ ^]^ 2H‐MoTe_2_ homojunctions.^[^ ^166^ ^]^

This review highlights the current research on next‐generation image sensors utilizing low‐dimensional semiconductor materials, emphasizing their potential in advanced imaging, biomimetic vision, and non‐von Neumann applications. The unique nanoscale structures of these materials confer exceptional electronic and optoelectronic properties, such as flexibility, tunable bandgaps, and strong light‐matter interactions, making them ideal for functional devices in next‐generation image sensors. The reported next‐generation image sensors, consisting of low‐dimensional materials (0D, 1D, 2D, and hybrid materials) in diverse device configurations for photodetection, showcase extensive imaging applications, as outlined in **Table** **1**. **Figure** **11**a summarizes the types of low‐dimensional materials, device structures, and pixels in the novel next‐generation image sensors. The PdS QDs are widely investigated as image sensors by integrating with silicon circuits, resulting in high resolution and photodiode structures. The relatively mature preparation technology of integrated 1D materials can realize the construction of high‐resolution image sensors. At present, the device structure consisting of nanowires is mainly concentrated on the two‐terminal photodetector structures. Currently, the research focus is on 2D materials and their heterostructures due to their superior optoelectronic properties, which have been constructed into photodetectors, photodiode, optical synapses, and transistors for imaging, showing great potential in biomimetic image sensors and non von Neumann imaging systems. Moreover, Figure 11b summarizes the responsivity and wavelength range of the single pixel in image sensors based on low‐dimensional materials. As can be seen, the responsivity of 2D heterojunctions consisting of WSe_2_/BN,^[^ ^53^ ^]^ CH_3_NH_3_PbI_3_/graphene,^[^ ^185^ ^]^ PbS/graphene^[^ ^20^ ^]^ reaches up to 10^7^ A/W, and most responsivity surpasses 1 A/W higher than that of the Si‐based image sensors. And the response range of PbS/HgTe QDs device is from 400 to 5000 nm,^[^ ^122^ ^]^ and type‐II Dirac semimetal PtTe_2_ has been utilized for room‐temperature terahertz photodetection with range from 0.02 to 0.3 THz.^[^ ^137^ ^]^ These sensors offer significant advantages over traditional 3D silicon‐based devices, making them suitable for intelligent imaging and artificial vision applications. With the development of low‐dimensional material preparation and integration technology, heterogeneous structures of various low‐dimensional materials with the combination of excellent properties will be the main materials of the next generation of image sensors to achieve high‐resolution, wide‐spectrum and high‐response imaging functions. In this following section, we explore the industry prototype and trends of next‐generation image sensors utilizing low‐dimensional materials, along with the challenges and future prospects for next‐generation integrated optoelectronics applications.

**Table 1:** A summary of image sensors based on low‐dimensional materials.

Types	Photosensitive materials	Pixels number	λ [nm]	*R* [A/W]	Device structure	Applications	Refs.
0D	PbS CQDs	256 × 256	1800	0.5	Photodiode	NIR imager	[121]
0D	PbS CQDs	640 × 480	1400	0.23	Photodiode	NIR imager	[120]
0D	PbS CQDs	640 × 512	400–1300	0.02‐0.5	Photodiode	NIR imager	[41]
0D	PbS and HgTe CQDs	640 × 512	400–5000	0.23‐0.83	Photodiode	Ultrabroadband imager	[122]
1D	Zn_2_GeO_4_ microwires	7 × 7	360	–	Lateral photodetector	Flexible imager	[22]
1D	CH_3_NH_3_PbI_3_ microwires	21 × 21	360–960	10.9‐13.57	Lateral photodetector	Flexible imager	[24]
1D	CH_3_NH_3_PbI_3_ nanowires	32 × 32	780	0.03	Vertical photodetector	Flexible imager	[23]
1D	Perovskite nanowires	10 × 10	400–810	0.002‐0.015	Electrochemical photodetector	Biomimetic eye	[45]
1D	Bi_2_Se_2_S nanowires	10 × 10	915–1550	0.37‐2.9	IR detection amplification system	Flexible NIR imager	[50]
2D	Silicon ribbons	16 × 16	620–700	–	Lateral photodetector	Biomimetic eye	[133]
2D	Silicon ribbons	16 × 16	620–700	–	Lateral photodetector	Biomimetic eye	[131]
2D	MoS_2_	900	450–625	10^4^	Phototransistor	In‐sensor de‐noising imager	[144]
2D	MoS_2_	5 × 5	550	–	Optical synapses	Biomimetic eye	[46]
2D	PtTe_2_	3 × 3	0.02–0.3 THz	0.074‐1.6	Lateral photodetector	Terahertz imager	[137]
2D	Graphene	48	440–700	4000	Phototransistor	Biomimetic eye	[188]
2D	CsPbBr_3_	10 × 10	350–530	1.2‐1.9	Lateral photodetector	Flexible imager	[52]
2D	CH_3_NH_3_PbBr_x_Cl_3‐x_	20 × 20	532	7	Vertical photodetector	Laser emission imager	[66]
2D	Bilayer MoS_2_	8 × 8	405–638	100‐119	Phototransistor	Highly sensitive imager	[69]
2D	CH_3_NH_3_PbI_3−x_Cl_x_	10 × 10	405–808	2.17	Lateral photodetector	Flexible imager	[147]
2D	Hydrophilic MoS_2_	10 × 10	480–700	–	Optical memory device	Optical memory imager	[167]
2D	MoO_x_	8 × 8	365	–	Vertical photodetector	Image machine learning	[54]
2D	Bilayer MoS_2_	8 × 8	660	–	Phototransistor	Image machine learning	[181]
0D+1D	ZnO QDs/Zn_2_SnO_4_ nanowires	10 × 10	300	3.6 × 10^6^	Lateral photodetector	Flexible UV imager	[151]
0D+1D	SnS QDs/Zn_2_SnO_4_	10 × 10	300–900	3‐10^5^	Lateral photodetector	Flexible NIR imager	[152]
0D+2D	PbS CQDs/InGaZnO	1	700–1400	150‐4 × 10^3^	Phototransistor	NIR imager	[155]
0D+2D	PbS CQDs/InGaZnO	1	1310	30‐10^4^	Phototransistor	NIR imager	[154]
0D+2D	PbS CQDs/graphene	388 × 288	300–2000	10^7^	Photodiode	Broadband imager	[20]
2D+2D	MoS_2_/graphene	1500	515–850	2	Phototransistor	Biomimetic eye	[159]
2D+2D	In_2_Se_3_/MoS_2_	10 × 10	490–1060	–	Optical synapse	Flexible NIR imager	[44]
2D+2D	CH_3_NH_3_PbI_3_/graphene	24 × 24	405–780	10^7^	Lateral photodetector	Flexible imager	[185]
2D+2D	Bi_2_Se_3_/Bi_2_Se_x_O_y_	1	405–1064	0.8–3	Vertical‐lateral photodetector	Broadband imager	[63]
2D+2D	InSe/WSe_2_/SnS_2_	1	400	0.035–0.55	Vertical photodetector	Self‐powered imager	[170]
2D+2D	GeSe_2_/GaN	4 × 4	205–400	0.01–0.26	Vertical photodetector	Self‐powered imager	[51]
2D+2D	WSe_2_/BN	3 × 9	473–638	1.2 ×10^7^	Optical memory device	Optical memory imager	[53]
2D+2D	2H‐MoTe_2_ homojunctions	10 × 10	520, 1060	0.0005–0.0006	Photodiode	Self‐powered imager	[166]

### 5.1 Prototype and Trend for Industry

Currently, CMOS image sensors (CIS) have become the dominant device in the industry due to their lower power consumption and cost‐effectiveness compared to conventional CCDs. The prevailing trends in the image sensor industry include the pursuit of enhanced sensitivity and high resolution, alongside miniaturization and improved energy efficiency. Additionally, there is a growing emphasis on the development of flexible and wearable sensors, which cater to the demands of modern applications. The integration of AI capabilities into image sensors is increasingly recognized as a critical factor in advancing their functionality and performance. These trends collectively reflect a significant evolution in the design and application of image sensors, aligning with contemporary technological advancements and user requirements. However, the further advancement of CIS is constrained by the low photoresponsivity (hundreds of mA·W*^−^* ^1^) and narrow wavelength range (visible to NIR wavelength) of silicon‐based photodetectors, along with issues like untunable photoresponsivity and mechanical rigidity, restricting their applications in biomimetic devices and intelligent imaging. The application of low‐dimensional materials for photosensitive units offers a promising avenue for creating the next‐generation image sensors that can enhance and potentially surpass current CIS technology to meet the trend of industry.

**Figure 12:**
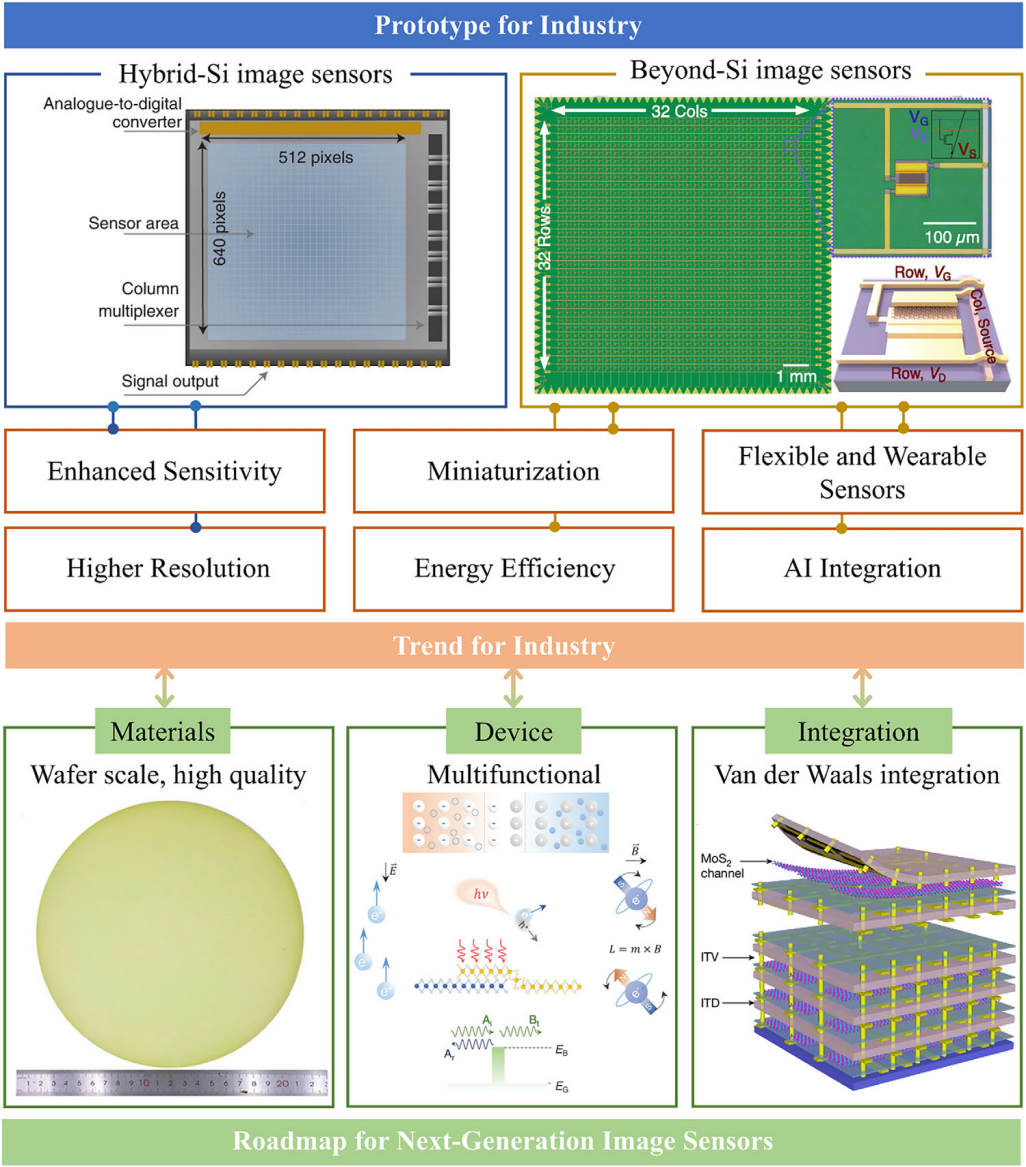
Roadmap for next‐generation image sensors based on low‐dimensional materials. Prototypes of image sensors for industry include hybrid‐Si image sensors and beyond‐Si image sensors. Reproduced with permission.^[^ ^41^ ^]^ Copyright 2022, Springer Nature Limited. Reproduced with permission.^[^ ^200^ ^]^ Copyright 2020, WILEY‐VCH Verlag GmbH. To realize the trend for industry in enhanced sensitivity, high resolution, miniaturization, energy efficiency, flexible and wearable sensors, AI integration, the low‐dimensional materials, devices, and integration techniques are required to be developed. Reproduced with permission.^[^ ^210^ ^]^ Copyright 2024, WILEY‐VCH Verlag GmbH. Reproduced with permission.^[^ ^211^ ^]^ Copyright 2024, WILEY‐VCH Verlag GmbH. Reproduced with permission.^[^ ^206^ ^]^ Copyright 2024, Springer Nature Limited.

There are two primary prototypes of image sensors based on low‐dimensional materials that are emerging in the industry. (**Figure** **12**) The first prototype is hybrid‐Si image sensors, which leverage the compatibility of low‐dimensional materials with traditional semiconductor fabrication processes to achieve enhanced sensitivity and high resolution. In contrast to conventional back‐illuminated CIS (BI‐CIS) technologies that rely on flip‐chip and wafer bonding methods, low‐dimensional materials can be transferred onto the surface of CMOS wafers under ambient temperature and pressure conditions. This capability facilitates feasible and efficient monolithic integration with silicon‐integrated circuits, thereby enabling the realization of BI‐CIS architectures. Furthermore, the expanding array of low‐dimensional materials offers a diverse library of materials, each possessing unique electrical and optoelectronic properties, which allows for the potential customization of image sensor functionalities to meet specific application requirements. For example, a NIR imager featuring 640 × 512 pixels has been constructed by combining PbS CQDs with a CMOS silicon readout integrated circuit.^[^ ^41^ ^]^ The optimized device structures yield a wide spectral range from 390 to 1300 nm, high responsivity (0.46 A∙W^−1^), and a *LDR* of 150 dB. The PbS CQD imager offers advantages in capturing detailed and informative images, which highlights the practical significance of PbS CQD imagers in various applications.

In the contemporary era of the IoT, the surge of unprocessed images has presented significant challenges to computing systems, as the frequent data shuffling between modules results in considerable power consumption. Furthermore, the technological disparity between memory and microprocessors exacerbates the physical separation between these components, leading to interconnect resistance mismatches and increased training latency. To mitigate these challenges, in‐memory computing utilizing resistive switching materials has emerged as a promising solution. This approach integrates memory and computing functions, thereby eliminating unnecessary data transfers between modules and fundamentally challenging the traditional von Neumann architecture. Such advancements illustrate how AI can enhance the physical implementation of conventional computing units. Notably, memristors, which leverage electrical channels to achieve multiple resistance states, facilitate substantial neural network workloads through efficient matrix computations. The second prototype is beyond‐Si image sensors, which capitalize on the unique physical properties of low‐dimensional materials, enabling the integration of sensing, memory, and computational functionalities within a single device to realize the integration of AI capabilities. Recently, inspired by biological vision, an analog optoelectronic processor with a crossbar array architecture consisting of 32 × 32 MoS_2_ phototransistors has been proposed.^[^ ^200^ ^]^ This image sensor emulates the eye and brain by capturing and storing optical images as electrical data and recognizing them through analog in‐memory neural network computing, achieving 94% accuracy in recognizing 1000 handwritten digits. Additionally, the distinctive mechanical properties of low‐dimensional materials facilitate the development of curved image sensors, thereby promoting miniaturization, energy efficiency, flexibility, and wearability.

### 5.2 Challenges and Perspectives

Despite significant advancements in the synthesis of low‐dimensional materials and the fabrication of advanced image sensors, practical applications in industry continue to encounter challenges related to materials, devices, and integration. These challenges encompass several critical areas, including the following points.

#### 5.2.1 Enhanced Sensitivity

Achieving enhanced sensitivity in image sensors is paramount for practical applications especially low‐light conditions. Recently, the integration of surface plasmon resonance (SPR) using metal nanoparticle arrays on low‐dimensional materials has significantly enhanced responsivity, such as Au nanoparticles for self‐powered InSe photodetector of 244 mA W^−1^ under visible illumination,^[^ ^39^ ^]^ or Al nanopaticles to increase the responsivity of graphene photodetectors under visible light by 25 times to 0.25 mA/W.^[^ ^201^ ^]^ Moreover, QD and CQD layer with high light absorption has been utilized for enhanced sensitivity. Graphene‐based photodetectors can achieve a responsivity of up to 10^7^ A/W in the near‐infrared range, significantly outperforming traditional silicon‐based sensors with hundreds of mA/W.^[^ ^20^ ^]^ However, the fabrication of SPR‐enhanced and QD‐based photodetectors are accompanied by issues including high dark currents and thermal noise, which degrade low‐light sensitivity. Additionally, the synthesis of metal nanopaticles and QDs involves complex and costly processes, restricting the scalability and integration with CMOS circuits of these technologies for image sensors. Addressing these challenges, it is essential to develop stable, uniform, and cost‐effective metal nanopaticles and QDs through optimal synthesis techniques to realize the stable integration processes with low‐dimensional semiconductor materials and circuit architectures. Additionally, other innovative strategies like construction of vertical‐lateral hybrid heterostructure^[^ ^63^ ^]^ or degisn of detection amplification system^[^ ^50^ ^]^ are needed to be further investigated to enhance sensitivity, reduce noise, and improve overall performance in next‐generation image sensors.

#### 5.2.2 High Resolution

High resolution is critical for the next‐generation image sensors in practical applications. Although low‐dimensional materials have the potential to improve pixel performance, significant challenges remain in scaling down pixel sizes without compromising image quality. Currently, 2D materials like MoS₂ have been used to fabricate 900‐pixel APS with individual phototransistors, achieving high‐resolution imaging with efficient noise reduction.^[^ ^144^ ^]^ However, the current pixel size of ≈10 µm in these devices is still larger than the 1–2 µm pixel size achievable in commercial CMOS sensors.^[^ ^202^ ^]^ Further development of fabrication and integration techniques, such as atomic layer deposition^[^ ^203^ ^]^ and through‐silicon via technology^[^ ^204^ ^]^ for low‐dimensional semiconductor materials are required to reduce pixel sizes and increase pixel numbers while maintaining or improving image quality.

#### 5.2.3 Device Miniaturization

The miniaturization of electronic devices necessitates the development of image sensors that can be integrated into increasingly compact systems. The optimization of integration between low‐dimensional materials and current semiconductor technology needs further research to refine fabrication methods and develop innovative designs that achieve highly miniaturized sensors without compromising functionality. For example, van der Waals heterostructures have been successfully integrated into curved image sensors with a truncated icosahedron layout, enabling high‐density imaging arrays without folds or wrinkles.^[^ ^159^ ^]^ However, challenges remain in achieving wafer‐scale integration of these materials, as current methods often result in defects and non‐uniformities that can degrade performance. Advanced fabrication techniques like electron‐beam lithography^[^ ^205^ ^]^ should be employed to enable precise patterning and integration of low‐dimensional materials at the nanoscale, while wafer‐scale integration^[^ ^14^ ^]^ and 3D stacking architectures^[^ ^206^ ^]^ can maximize spatial efficiency and reduce device footprint.

#### 5.2.4 Energy Efficiency

Energy efficiency is a critical consideration in the design of image sensors, particularly for portable and battery‐operated devices. Recently, self‐powered image sensors with ultralow power consumption have been constructed by designing built‐in electric fields in devices consisting of low‐dimensional materials. For instance, 2H‐MoTe₂ homojunctions have been used to create self‐powered image sensors under near‐infrared illumination without external bias.^[^ ^166^ ^]^ These devices show great potential in energy‐efficient imaging, but further optimization is needed to improve their responsivity and dynamic range. To enhance energy efficiency in next‐generation image sensors, researchers should focus on optimizing self‐powered designs using low‐dimensional materials^[^ ^85^ ^]^ by improving built‐in electric fields and carrier dynamics to boost responsivity and dynamic range. Additionally, integrating energy‐harvesting technologies, like solar cells^[^ ^207^ ^]^ or piezoelectric materials,^[^ ^40^ ^]^ with image sensors can further reduce power consumption, enabling sustainable operation in portable and battery‐operated devices.

#### 5.2.5 Flexible and Wearable Technology

The demand for flexible and wearable technology is growing rapidly, requiring that next‐generation image sensors can conform to various surfaces and withstand mechanical stress. Although low‐dimensional materials exhibit inherent flexibility and lightweight nature, challenges persist in ensuring durability and performance under diverse conditions. For example, ultrathin perovskite films have been used to fabricate 10 × 10 pixel arrays that are both flexible and lightweight, with a total thickness of only 1.1 µm.^[^ ^52^ ^]^ These devices have demonstrated excellent mechanical properties, including the ability to be compressed on human skin without delamination. However, long‐term durability under repeated mechanical stress remains a challenge, and further research is needed to optimize material properties and device architectures for robust, high‐performance flexible image sensors tailored to wearable applications. Developing direct synthesis method^[^ ^182^ ^]^ on flexible substrates and a sophisticated transfer method^[^ ^188^ ^]^ shows potentials to achieve flexible and wearable image sensors based on low‐dimensional materials. Additionally, exploring hybrid material systems and innovative device architectures, such as stretchable interconnects^[^ ^208^ ^]^ and strain‐resistant substrates,^[^ ^209^ ^]^ can further improve the long‐term performance and reliability of flexible image sensors for wearable applications.

#### 5.2.6 AI Integration

The integration of AI into image sensor technology represents a transformative opportunity to enhance functionality and enable real‐time data processing. However, effectively embedding AI algorithms within the sensor architecture poses significant challenges, including computational efficiency, power consumption, and hardware‐software co‐design. For example, MoS₂‐based ORRAM devices have demonstrated neuromorphic computing capabilities, achieving 94% accuracy in recognizing handwritten digits by integrating sensing, memory, and processing functions within a single device.^[^ ^54^ ^]^ Similarly, graphene‐based photodetectors with integrated AI algorithms have shown potential for in‐sensor computing, reducing data transfer latency and power consumption by processing information directly at the sensor level.^[^ ^20^ ^]^ Developing energy‐efficient neuromorphic architectures, optimizing in‐memory computing techniques, and leveraging low‐dimensional materials with tunable electronic properties to enable seamless AI integration have attracted increasing attention. The integration of AI and image sensor technology can unlock new possibilities for intelligent imaging systems in applications ranging from autonomous vehicles to biomedical diagnostics.

Nevertheless, low‐dimensional materials have not yet reached a level of practical application in image sensors. Several technical challenges remain to be resolved, including the development of synthesis methods for stable and wafer‐scale single crystals, the design and fabrication of multifunctional devices, and the advancement of integration techniques for heterogeneous low‐dimensional semiconductor materials. Figure 12 shows the roadmap for next‐generation image sensors consisting of low‐dimensional semiconductor materials. The synthesis of wafer‐scale and high‐quality low‐dimensional semiconductor materials is a current research hotspot in the field of materials and chemistry. Recently, 8‐in. wafer‐scale monolayer MoS_2_ films with excellent spatial homogeneity have been epitaxially synthesized on sapphire through a designed vertical CVD system.^[^ ^210^ ^]^ As the novel low‐dimensional semiconductor materials and nano fabrication techniques continuously developing, breakthrough progress has been made in multifunctional and high‐performance photoelectric conversion devices.^[^ ^211^ ^]^ Apart from the optical modulation and electric field, the magnetic field and quantum states are induced in these image sensors consisting of low‐dimensional materials, accelerating the development of biomimetic vision system and non von Neumann imaging systems. Furthermore, the dangling‐bonds‐free surface of most low‐dimensional materials overcomes the limitations of lattice matching, providing advantages for monolithic 3D integration on various substrates. Van der Waals lamination shows great potential in the hybrid‐Si image sensors and beyond‐Si image sensors.^[^ ^206^ ^]^ Monolithic 3D integrated systems are constructed by repeating the van der Waals lamination process tier by tier in the vertical direction, which will be further developed and applied in the next‐generation image sensors. In summary, the advancement of low‐dimensional materials, coupled with ongoing innovations in device architectures and integration techniques, is essential for the development of next‐generation image sensors. This breakthrough in the imaging field necessitates interdisciplinary collaboration and cross‐domain exchanges among researchers in materials, chemistry, physics, semiconductor, and artificial intelligence.

## Conflict of Interest

The authors declare no conflict of interest.
